# Repurposing effect of cardiovascular-metabolic drug to increase lifespan: a systematic review of animal studies and current clinical trial progress

**DOI:** 10.3389/fphar.2024.1373458

**Published:** 2024-06-20

**Authors:** Agian Jeffilano Barinda, Harri Hardi, Melva Louisa, Nurul Gusti Khatimah, Rheza Meida Marliau, Immanuel Felix, Muhamad Rizqy Fadhillah, Arief Kurniawan Jamal

**Affiliations:** ^1^ Department of Pharmacology and Therapeutics, Faculty of Medicine, Universitas Indonesia, Jakarta, Indonesia; ^2^ Metabolic, Cardiovascular, and Aging Cluster, Indonesia Medical Education and Research Institute (IMERI), Faculty of Medicine, Universitas Indonesia, Jakarta, Indonesia; ^3^ Master Program in Biomedical Sciences, Faculty of Medicine, Universitas Indonesia, Jakarta, Indonesia; ^4^ Division of Endocrinology, Metabolism, and Diabetes, Department of Internal Medicine, Dr. Cipto Mangunkusumo National General Hospital, Faculty of Medicine Universitas Indonesia, Jakarta, Indonesia

**Keywords:** aging, drug repositioning, cardiovascular, metabolic, lifespan, animal model, clinical trial

## Abstract

With the increase in life expectancy, aging has emerged as a significant health concern. Due to its various mechanisms of action, cardiometabolic drugs are often repurposed for other indications, including aging. This systematic review analyzed and highlighted the repositioning potential of cardiometabolic drugs to increase lifespan as an aging parameter in animal studies and supplemented by information from current clinical trial registries. Systematic searching in animal studies was performed based on PICO: “animal,” “cardiometabolic drug,” and “lifespan.” All clinical trial registries were also searched from the WHO International Clinical Trial Registry Platform (ICTRP). Analysis of 49 animal trials and 10 clinical trial registries show that various cardiovascular and metabolic drugs have the potential to target lifespan. Metformin, acarbose, and aspirin are the three most studied drugs in animal trials. Aspirin and acarbose are the promising ones, whereas metformin exhibits various results. In clinical trial registries, metformin, omega-3 fatty acid, acarbose, and atorvastatin are currently cardiometabolic drugs that are repurposed to target aging. Published clinical trial results show great potential for omega-3 and metformin in healthspan.

**Systematic Review Registration:** crd.york.ac.uk/prospero/display_record.php?RecordID=457358, identifier: CRD42023457358.

## 1 Introduction

Aging is a complex and inexorable process that correlates with a decrease in capability status and physiological functions thus eventually leading to the amelioration of healthspan and shortening of lifespan. Growing evidence showed that aging was found to be an irreversible risk factor for multiple comorbid, including diabetes (Chentli et al., 2015), cardiovascular disease (North and Sinclair, 2012; Rodgers et al., 2019), neurodegenerative disease (Xia et al., 2018), and cancer (Berben et al., 2021). Global Burden of Disease Study revealed that the mortality rates were higher in older adult populations since various degenerative diseases have been detected in these populations (Roth et al., 2018).

The recent innovation of medical science has greatly empowered our understanding of the molecular mechanisms of aging and developed new potential approaches for deferring the aging process (Campisi et al., 2019). Numerous aging interventions, including gerotherapeutics were shown to increase lifespan and prevent the occurrence of chronic disorders linked to aging (Partridge et al., 2020).

Cardiovascular and metabolic drugs are frequently repurposed due to their diverse molecular mechanism in many diseases (Ishida et al., 2016; Schubert et al., 2020). With various molecular mechanisms found in the aging process, cardiometabolic drugs possess the potential to delay aging. For instance, aspirin and statin are potentially beneficial for cancer (Zaleska et al., 2018; Ahmadi et al., 2020; Wang et al., 2021), or the pleiotropic effect of metformin in cancer, cardiovascular disease, and dementia in diabetic patients (Barzilai et al., 2016). Of note, aspirin and metformin could extend the lifespan of rodents (Strong et al., 2008; Martin-Montalvo et al., 2013). However, other reviews usually focus on *in vitro* scoring, 3D protein structures, orthology relationship, and drug binding, all of which require additional validation through *in vivo* study and clinical trial (Ziehm et al., 2017; Dönertaş et al., 2018). Therefore, our systematic review primarily focused on animal studies, with additional consideration given to clinical trials and their protocols.

Dramatic growth in the variety of longevity medicines that are being identified from animal studies is not always successfully translated to clinical applications (de Magalhães, 2021). Aspirin treatment failed to prevent mortality and morbidity in healthy older adult people and potentially increased the hemorrhagic risk in those people (McNeil et al., 2018b; 2018a). In parallel, metformin could not prolong the lifespan in *drosophila* and rather increased the mortality in female mice (Slack et al., 2012; Anisimov et al., 2015). Moreover, the clinical trials of metformin, such as MILES (Metformin In Longevity Study), showed the enhancement of longevity-related gene expressions, but the valid molecular mechanisms by which metformin facilitates this activity remain unknown (Mohammed et al., 2021).

This systematic review will summarize and analyze the evidence of cardiovascular and metabolic drugs from pre-clinical animal studies and recent clinical trials and highlight the rationale for the use of the repurposing potential of cardiometabolic drugs to increase lifespan in animal studies.

## 2 Materials and methods

This systematic review was conducted in accordance with the Preferred Reporting Items for Systematic Reviews and Meta-Analysis (PRISMA) guidelines (Page et al., 2021). The study protocol can be observed on The International Prospective Register of Systematic Reviews (PROSPERO) database: https://www.crd.york.ac.uk/prospero/display_record.php?RecordID=457358. This systematic review of animal studies aims to determine the effect size and mechanism underlying lifespan increase. Following this, a search was conducted on the ICTRP (International Clinical Trial Registry Platform) clinical trial registry using the identified cardiometabolic drug from the animal studies.

### 2.1 Study eligibility criteria

We selected all interventional animal studies that met specific inclusion and exclusion criteria for our study using the PICO framework. The P stands for “animal,” I for “cardiometabolic drugs,” C for “no cardiometabolic drug,” and O for “lifespan.” Any animal model (natural or gene-modified animal to induce aging) is included in the Population. To replicate the natural aging process in humans, we exclude any intervention or induction that induces any disease apart from aging throughout the animal’s lifetime. Yeast lifespan studies were also excluded because they are not a proper model for human aging studies (Zadrag et al., 2008).

As for intervention, we include all healthy animals who are given any routine cardiometabolic drug as part of the intervention at any time of their life until the animal is dead. Cardiometabolic drugs that are not currently approved by the FDA (Food and Drug Administration) or stated in the AHA (American Heart Association) and ADA (American Diabetes Association) cardiometabolic drug list are excluded (AHA, 2020; 2023; ADA, 2023). Any intervention during animal life that can cause a difference in their lifespan, such as an unnatural diet, is also excluded. We also exclude the comparator other than placebo because it will be a source of bias. Treatment other than intervention should be the same.

The primary outcome of this study is median or mean lifespan. In the absence of median or mean lifespan information, we would still consider including an article on cardiometabolic drugs if it included a Kaplan-Meier curve. The secondary outcome in this systematic review is healthspan, which consists of cardiometabolic, neurodegenerative, musculoskeletal, and neoplasm outcomes. Any other outcome that will impact animal health is also included. We also include any laboratory parameters that relate to lifespan and healthspan.

### 2.2 Search strategy

We conducted animal systematic literature search based on PICO “animal”, “cardiometabolic drugs”, and “lifespan”. Each cardiometabolic drug was searched individually based on the AHA and ADA cardiometabolic drug lists (AHA, 2020; 2023; ADA, 2023). Our complete search strategies from four databases (Pubmed, Embase, Web of Science, and Scopus) are detailed in Supplementary Table S1. We considered all animal studies regardless of language and year of publication.

Clinical trial registries in lifespan and healthspan were also searched in ICTRP (International Clinical Trials Registry Platform). We considered conducting a search at ICTRP for all cardiometabolic drugs found in animal trials. Our search strategies consist of “cardiometabolic drug name” and “aging”. Completed clinical trial registries are manually searched for the full text. All clinical trial results will be described as a narrative review.

### 2.3 Study selection

**FIGURE 1:**
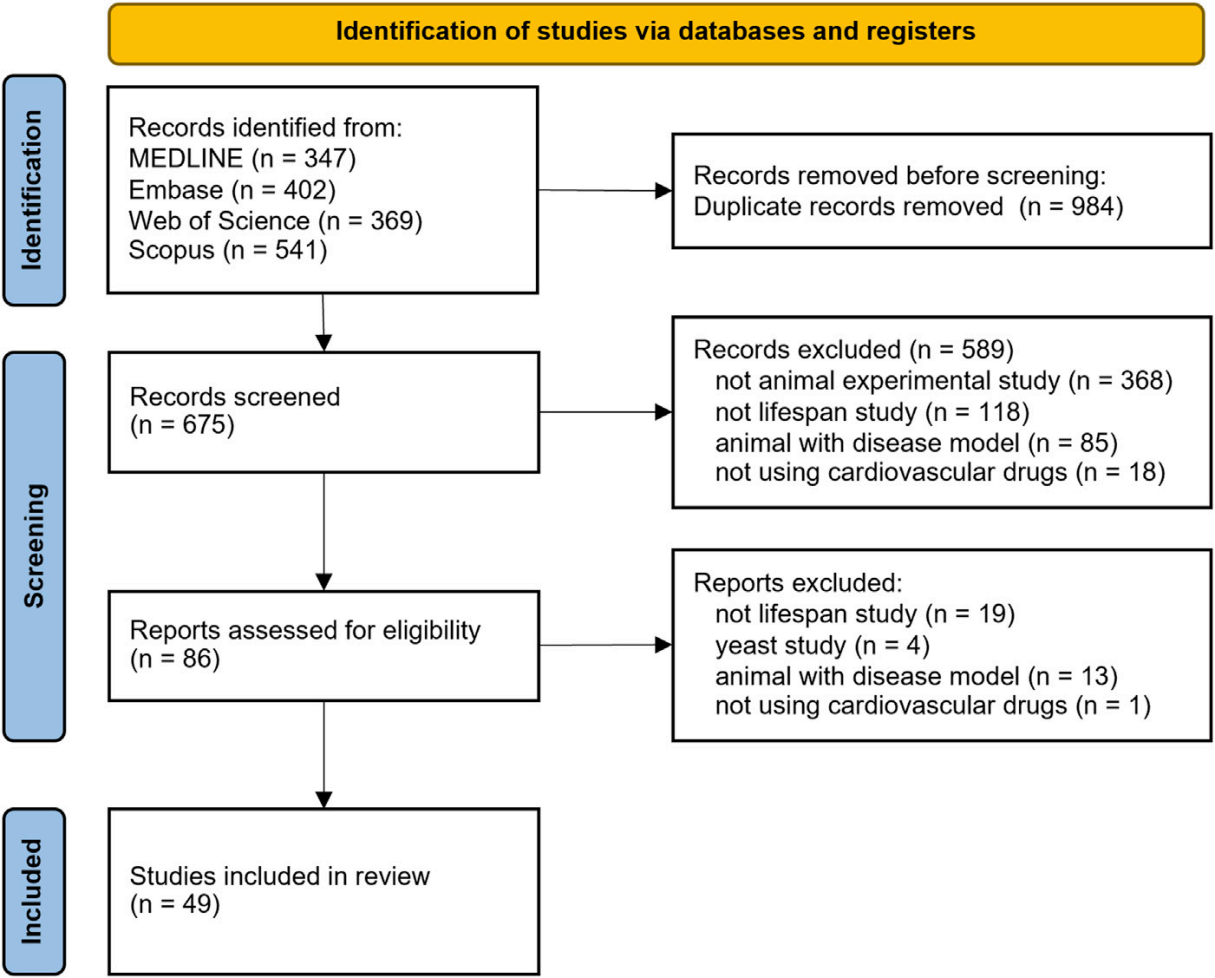
PRISMA flow chart search strategy.

Based on our search, all found studies are collected and managed in Mendeley Desktop version 1.19.8 (Glyph & Cog LLC, 2020). The software will automatically delete any duplicates. Furthermore, we manually identified and excluded other duplicates that cannot be detected by the software. Two independent authors (HH and AJB) screened all non-duplicate titles and abstracts according to inclusion and exclusion criteria; further discrepancies were discussed with a third author (ML). We recorded all reasons for excluded records as outlined in Figure 1.

We obtained all full text of included studies based on title and abstract screening by searching or buying the full text. Unobtainable full text was requested from the corresponding author. We excluded the unobtainable full text if the corresponding author did not respond. All full text eligibility was evaluated by two independent authors (HH and AJB) in accordance with inclusion and exclusion criteria; any discrepancies were resolved through consultation with a third author (ML).

### 2.4 Data extraction and management

All included animal study data were obtained based on PICO:

• Method: study design, year of study, number of study locations
• Animal model: species, gender, species strain/gene-modification, total animal used
• Intervention: drug name, dose, age at treatment initiation, comparator (placebo)
• Outcome: primary and secondary outcome
• Notes: funding and conflict of interest of the study

Two authors (HH and AJB) individually extracted the data and other potential data related to the results. We resolved the disagreement by consensus with the third author (ML). The results of this consensus were input to a word processor, and another author (NGK) double-checked all data input. If any changes were made, the other first three authors were asked about the appropriateness.

### 2.5 Risk of bias assessment

We used SYRCLE’s risk of bias tool for animal studies to assess the risk of bias in this study (Hooijmans et al., 2014). This tool consists of six domains (selection, performance, detection, attrition, reporting, and other bias) and ten questions based on animal intervention study potential of bias. We also used RoB 2 tool for assessing the risk of bias in published clinical trial registry studies (Sterne et al., 2019). Three independent authors (HH, AJB, and NGK) individually searched and discussed all potential biases of all included studies.

### 2.6 Measures of treatment effect

This systematic review of treatment effect is based on its primary outcome, lifespan. Lifespan in animal studies is commonly stated as the increase in percentage compared to control. We do not intend to proceed with a meta-analysis of this systematic review due to the numerous heterogeneities present in the study, including different cardiometabolic drugs, drug dosages, and animal models. As a result, meta-analysis is deemed unsuitable for the present study design.

## 3 Results

A comprehensive search across four databases yielded a total of 675 studies. Search results for each cardiometabolic drug can be seen in Supplementary Table S2. We identified 49 studies after applying the inclusion and exclusion criteria outlined in the methods section. The complete PRISMA flowchart for this study is illustrated in Figure 1. All included animal trials were evaluated based on PICO.

**FIGURE 2:**
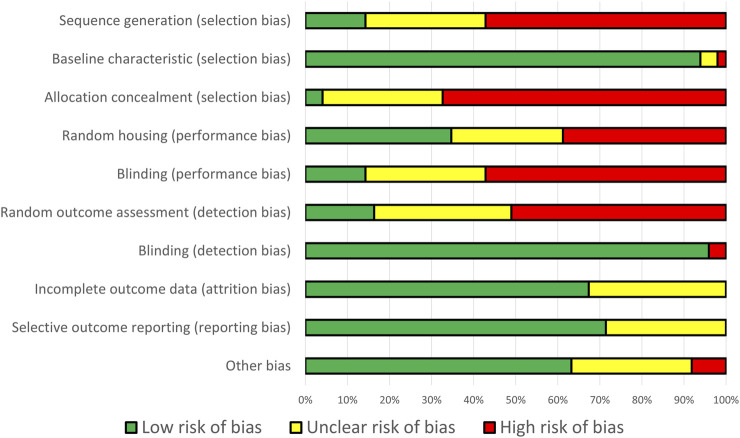
Percentage of bias risk across all domains from 49 identified studies.

Sequence generation, allocation concealment, random housing, blinding, and random outcome assessment were all found to have a significant risk of bias. Although random housing is impractical for smaller animals, concealment and blinding are critical for animal research. Nevertheless, the potential for bias in these studies could be mitigated because lifespan is an objective parameter. Additional operator-dependent healthspan parameters, such as muscle size, may lead to bias in the absence of adequate blinding and concealment. The comprehensive RoB assessment of each study is detailed in Supplementary Table S3. The summary of RoB result is illustrated in Figure 2.

A diverse range of animals, including rats, mice, common fruit flies, roundworms, and silkworms, were utilized in these studies. Drug exposure starts at various stages of life in animals. Prolonged drug exposure yields more favorable results regarding extending lifespan (Espada et al., 2020; Strong et al., 2022).

Diverse drug concentrations also exhibit distinct impacts on the extension of lifespan. Research on captopril and metformin has demonstrated that while an appropriate dose of cardiometabolic drugs appears to extend lifespan, higher doses can shorten it (Martin-Montalvo et al., 2013; Anisimov et al., 2015; Onken et al., 2022; Egan et al., 2023). Our full list of extraction data can be seen in Supplementary Table S4. We summarized the data in Table 1.

**TABLE 1:** Summary of animal trials finding.

Drug(s)	Study found	References
Cardiovascular drugs		
Acetazolamide	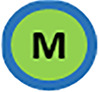	Leibrock et al. (2016)
Aspirin	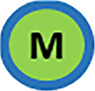 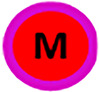 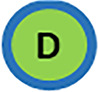 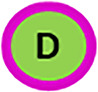 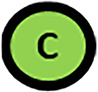 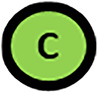 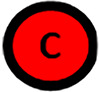	Strong et al. (2008), Strong et al. (2008), Ayyadevara et al. (2013), Huang et al. (2013), Wan et al. (2013), Danilov et al. (2015), Danilov et al. (2015)
Candesartan	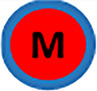 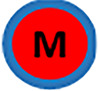 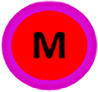	Spindler et al. (2016), Harrison et al. (2021), Harrison et al. (2021)
Captopril	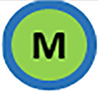 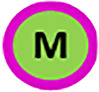 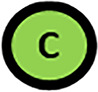 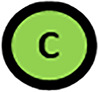	Kumar et al. (2016), Strong et al. (2022), Strong et al. (2022); Egan et al. (2023)
Enalapril	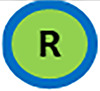	Santos et al. (2009)
Hydralazine	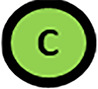 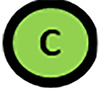	Dehghan et al. (2017), Dehghan et al. (2019)
Metolazone	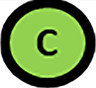	Ito et al. (2021)
Metoprolol	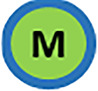 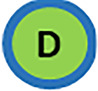	Spindler et al. (2013), Spindler et al. (2013)
Nevibolol	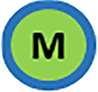 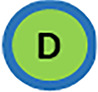	Spindler et al. (2013), Spindler et al. (2013)
Ramipril	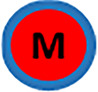	Spindler et al. (2016)
Verapamil	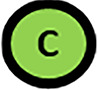	Liu et al. (2020)
Antidiabetic drugs		
Acarbose	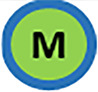 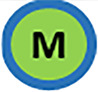 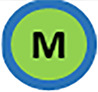 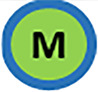 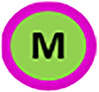 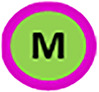 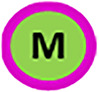 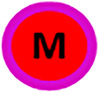 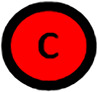	Harrison et al. (2014), Harrison et al. (2014), Strong et al. (2016); Strong et al. (2016), Harrison et al. (2019), Harrison et al. (2019); Smith et al. (2019), Smith et al. (2019), Banse et al. (2023)
Canaglifozin	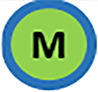 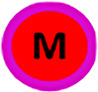	Miller et al. (2020), Miller et al. (2020)
Dapaglifozin	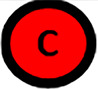	Onken et al. (2022)
Glibenclamide	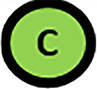	Mao et al. (2022)
Glimepiride	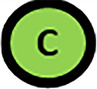	Mao et al. (2022)
Glipizide	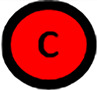	Onken et al. (2022)
Linagliptin	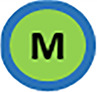	Hasegawa et al. (2017)
Metformin	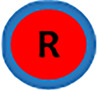 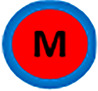 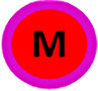 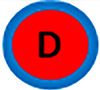 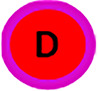 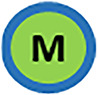 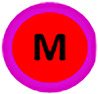 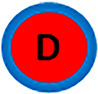 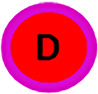 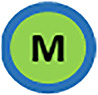 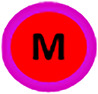 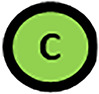 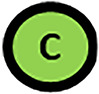 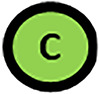 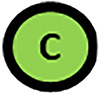 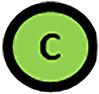 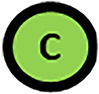 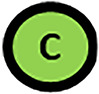 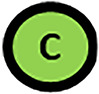 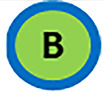 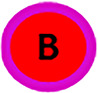	Onken and Driscoll (2010), Smith et al. (2010), Slack et al. (2012), Slack et al. (2012), Cabreiro et al. (2013), Martin-Montalvo et al. (2013), De Haes et al. (2014), Anisimov et al. (2015), Anisimov et al. (2015), Strong et al. (2016), Strong et al. (2016), Chen et al. (2017), Abrat et al. (2018), Abrat et al. (2018); Song et al. (2019), Song et al. (2019), Espada et al. (2020), Zhu et al. (2021), Onken et al. (2022), Xiao et al. (2022), Cedillo et al. (2023)
Nateglinide	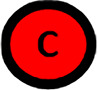	Onken et al. (2022)
Pioglitazone	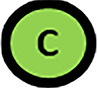 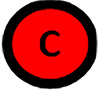	Jia et al. (2022), Onken et al. (2022)
Rosiglitazone	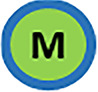	Xu et al. (2020)
Sitagliptin	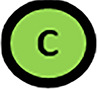	Onken et al. (2022)
Dyslipidemia drugs		
Clofibrate	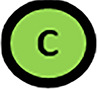	Brandstädt et al. (2013)
Fenofibrate	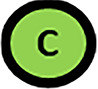	Brandstädt et al. (2013)
Lovastatin	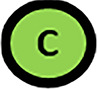	Andreas et al. (2020)
Niacin	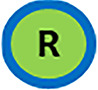 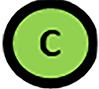	Preuss et al. (2011), Yang et al. (2019)
Omega-3 PUFA	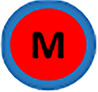 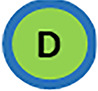	Spindler et al. (2014), Champigny et al. (2018)
Simvastatin	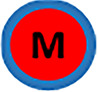 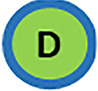	Spindler et al. (2012), Spindler et al. (2016)
Drugs combination		
Acarbose + Rapamycin	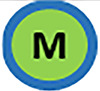 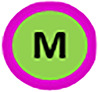	Strong et al. (2022), Strong et al. (2022)
Metformin + Rapamycin	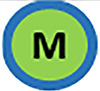 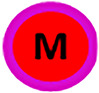	Strong et al. (2016), Strong et al. (2016)
Ramipril + Simvastatin	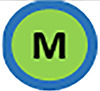	Spindler et al. (2016)

One circle indicates one study result; a study with various doses is denoted as one circle. R: rat (*Rattus norvegicus*), M: mouse (*Mus musculus*), B: silkworm (*Bombyx morii*), C: roundworm (*Caenorhabditis elegans*, *Caenorhabditis brigisae*, or *Caenorhabditis tropicalis*), D: common fruit fly (*Drosophila melanogaster*). The blue border indicates male, pink indicates female, and black indicates hermaphrodite roundworm. Green fill indicates a positive effect in increasing lifespan, whereas red fill indicates no effect in increasing lifespan. References are consecutively arranged according to the circle.

### 3.1 The lifespan extension effect of cardiovascular drugs

Nine cardiovascular drugs in prolonging lifespan (acetazolamide, aspirin, captopril, enalapril, hydralazine, metolazone, metoprolol, nebivolol, and verapamil) were found to extend lifespan significantly, while the other two (candesartan and ramipril) did not show the same effect. Some drugs (hydralazine, metolazone, and verapamil) only have been tested in *Caenorhabditis elegans*. Therefore, further higher animal studies are needed.

Aspirin was successfully shown as a lifespan-extending compound in *C. elegans*, *drosophila*, and mice (Ayyadevar et al., 2013; Danilov et al., 2015; Strong et al., 2008; Wan et al., 2013). However, one study found that aspirin failed to extend the lifespan in *C. elegans* with glp-1 mutation (Huang et al., 2013). An additional interesting discovery pertains to the fact that certain cardiovascular drugs within the same class, ACE inhibitors (ACE-I), exhibit distinct characteristics in terms of prolonging lifespan. Ramipril lacks the ability to induce an extension in lifespan, but not in captopril and enalapril (Santos et al., 2009; Kumar et al., 2016; Spindler et al., 2016; Egan et al., 2023). Meanwhile, candesartan as Angiotensin Receptor Blocker (ARB) failed to extend the lifespan in mice (Harrison et al., 2021).

### 3.2 The lifespan extension effect of dyslipidemia drugs

Many drugs that aim to increase lifespan and are linked to dyslipidemia have been tested on different organisms. Niacin (nicotinic acid) was discovered to increase the lifespan of *C. elegans* at a concentration of 600 nmol, but not at 100 and 200 nmol (Yang et al., 2019). It also extended the lifespan of Zucker Fatty rats (Preuss et al., 2011). Simvastatin has been shown to increase lifespan in *Drosophila* but not in mice (Spindler et al., 2012; 2016). Furthermore, the combination of simvastatin and ramipril extended the lifespan of mice (Spindler et al., 2016). Lovastatin also extended the lifespan of *C. elegans* (Andreas et al., 2020). Fenofibrate increased lifespan in a dose-dependent manner, while clofibrate only extended lifespan at a concentration of 10 µM in *C. elegans* (Brandstädt et al., 2013). An interesting study on omega-3 found that it significantly extends the lifespan of *Drosophila* but appears to reduce the lifespan of mice, although the result was not statistically significant (Spindler et al., 2014; Champigny et al., 2018). The results highlight that the lifespan extension effects of cardiometabolic drugs vary depending on the species.

### 3.3 The lifespan extension effect of antidiabetic drugs

Anti-diabetic medications were the most used drugs repurposed for aging. Three antidiabetic medications (acarbose, canagliflozin, and rosiglitazone) have been found to significantly extend lifespan, with acarbose being the most extensively researched. Positive effects are predominantly seen in male animals, whereas research has been unsuccessful to prolong lifespan in females. Additionally, metformin was found to be insignificant in rat (Smith et al., 2010) and beneficial in only two out of six rodent studies (Cabreiro et al., 2013; Zhu et al., 2021). Every study conducted on metformin in *C. elegans* is significant. It demonstrates that the effect of metformin on life expectancy varies by species. Metformin study in various *Caenorhabditis* species also showed that metformin’s beneficial effects on lifespan are limited to *C. elegans* but not in *Caenorhabditis briggsae* or *Caenorhabditis tropicalis* (Onken et al., 2022).

### 3.4 The lifespan extension effect of drug combination

Several studies employing drug combinations, including ramipril and simvastatin, acarbose and rapamycin, and metformin and rapamycin, were identified. Ramipril or simvastatin alone do not increase lifespan in mice, but the combination of these medications significantly extends lifespan Simvastatin may blunt insulin sensitivity, while both simvastatin and ramipril induce hypercholesterolemia and hypertriglyceridemia (Spindler et al., 2016). A significant increase in lifespan has also been observed when rapamycin is combined with metformin or acarbose. We cannot ascertain whether these interactions are additive or synergistic, but it is speculated that these anti-diabetics may prevent hyperglycemia due to rapamycin administration by enhancing insulin sensitivity (Strong et al., 2016; 2022). Of note, rapamycin has been shown in a meta-analysis study of laboratory mice that may significantly increase the lifespan (Swindell, 2016). In summary, drug combination trials may be regarded as prospective areas of research in the field of lifespan.

### 3.5 Completed and ongoing clinical trial of cardiometabolic drug in aging

We identified 14 of the 44 study registries discovered in ICTRP that met our inclusion and exclusion criteria. We obtained data from these registries on 12 healthy elderly individuals and 2 patients with HIV. Search result is detailed in Supplementary Table S5. We discovered clinical trial registries for six metformin, four omega-3 fatty acids, two acarbose, one fenofibrate, and one atorvastatin. The results of the ten registries are detailed in Table 2, of which results for four have been published. We put the other four registries in Supplementary Table S6 due to unknown, terminated, or withdrawn status. One study was terminated due to recruitment being difficult and not achieved.

**TABLE 2:** Current completed and ongoing clinical trial in repurposing cardiometabolic drug for aging.

Clinical trial identifier	Year of registration	Study name	Drug name (dose)	Drug administration length	Total subject (enrollment)	Total subject (finished)	Participant age	Availability of result
NCT00996229	2009	Effects of Dietary Interventions on the Aging Brain	Omega 3 (2.2 g/d)	26 weeks	Exp: 40 Con: 40	Exp: 22 Con: 27	50–75	Yes, (Külzow et al., 2016)
NCT02102724	2014	Fish Oil for HIV-Related Inflamm-aging and Immune Senescence	Omega 3 (1.6 g/d)	12 weeks	Exp: 18 Con: 19	Exp: 16 Con: 18	40–70	Yes, (Swanson et al., 2018)
NCT02953093	2016	Study of acarbose in Longevity (SAIL)	Acarbose (no data in dose)	10 weeks	Crossover trial: 10	NA	60–100	No
NCT02865499	2016	Acarbose Anti-aging Effects in Geriatric Subjects (Substudy B & C)	Acarbose (300 mg/d)	8 weeks	Pre and post-study: 8	6	70–95	Yes, available on https://clinicaltrials.gov/study/NCT02865499
NCT04386577	2017	Effects of Vitamin D and Omega-3 Supplementation on Telomeres in VITAL	Omega 3 (840 mg/d)	208 weeks	Exp: 250 Con: 250	NA	Men > 50 Women >55	No
NCT03228550	2017	Omega-3 Fatty Acids and Exercise on Mobility and Cognition in Older Women (MOBILE)	Omega 3 (1.16 g/d)	24 weeks	Exp: 15 Con: 15	Exp: 12 Con: 13	>60	Yes, (Fairbairn et al., 2020)
NCT02432287	2018	Metformin in Longevity Study (MILES)	Metformin (1.7 g/d)	6 weeks	Crossover trial: 16	14	>60	Yes, (Kulkarni et al., 2018)
NCT04264897	2020	Antecedent Metabolic Health and Metformin Aging Study (ANTHEM)	Metformin (1.5 g/d)	12 weeks	Exp: 74 Con: 74	NA	40–75	No, protocol published at (Kumari et al., 2022)
NCT04536870	2020	Statins in Reducing Events in the Elderly (STAREE) Heart Sub-study (STAREE-HEART)	Atorvastatin (40 mg/d)	162 weeks	Exp: 500 Con: 500	NA	>70	No, protocol published at (Zoungas et al., 2023)
EUCTR 2021-003299-15-ES	2021	Metformin vs. placebo for reversal of accelerated biological aging in persons living with HIV 50 years	Metformin (850 mg/d)	96 weeks	Exp: 60 Con: 60	NA	>50	No

Dose of omega 3: 1,320 mg EPA and 880 mg DHA (Külzow et al., 2016); 800 mg EPA, 600 mg DHA, and 200 mg other omega-3 fatty acids (Swanson et al., 2018); 465 mg EPA and 375 DHA; dietary supplements (1,000 mg DHA, 160 mg EPA, 1 mg folic acid, 124 phosphatidylserine, 240 mg *G. biloba*) (Fairbairn et al., 2020). Doses are arranged respectively based on all omega-3 studies. Exp: experimental total subject. Con: control total subject.

We identified three published clinical trial results in omega-3 (Külzow et al., 2016; Swanson et al., 2018; Fairbairn et al., 2020) and one in metformin (Kulkarni et al., 2018). Supplementary Table S7 shows a summary of clinical trials study risk of bias. In one study, the proportion of smokers in the control group was significantly greater than in the experimental group (Swanson et al., 2018). The other study has a dropout rate of over 20%, which lowers its significance result (Külzow et al., 2016).

## 4 Discussion

The breakthrough of gerotherapeutic as medication that molecularly targets the aging has become an emerging new era for overcoming shortened lifespans and preventing age-related pathologies (Couteur and Barzilai, 2022). Moreover, cardiovascular and metabolic pharmacology have been evaluated as candidates for gerotherapeutics in both preclinical and clinical models (Williams and Kim, 2003; Barzilai et al., 2016).

This systematic review has compiled the lifespan extension effect of cardiovascular and metabolic pharmacological interventions in animal models. The animal models, particularly in rodents, with human pathology phenotypes and yeast models were excluded from this study to maintain the quality of this review. The gold standard of drug identification with lifespan extension study in rodent models has been reviewed elsewhere (Spindler, 2012). As mentioned in this review, long-lived and healthy rodents, such as F1 hybrid mice, are ideally recommended for longevity drug screening. Several drugs that successfully extended the lifespan were mostly reported in short-lived or pathological mice models such as obese or diabetic mice. The failure of reproductivity data of these lifespan extension compounds in healthy rodents is likely due to the consequence of using pathological rodent models. Therefore, we did not include those models in this systematic review. We also excluded the lifespan studies that used yeast (*Saccharomyces cerevisiae*) since this model is not an appropriate model for representing aging in humans (Zadrag et al., 2008).

### 4.1 Cardiovascular drugs

The presence of chronic and low-grade inflammation phenotype is strongly associated with the process of aging. An *in vivo* study using a chronic inflammation mice model demonstrated accelerated aging and reduced regeneration capacity in the mice (Jurk et al., 2014). Moreover, anti-inflammation therapy such as Non-Steroid Anti-Inflammatory Drugs (NSAID) prevents the senescence phenotype. Additionally, Cyclooxygenase 2 (COX-2) and Prostaglandin E2 (PGE2) were identified to be involved in inflammation-mediated senescence (Martien et al., 2013). These data suggested the potential role of aspirin in delaying the aging process.

This systematic review summarized that aspirin was well conserved as a lifespan-extending compound in *C. elegans*, *drosophila*, and mice (Ayyadevar et al., 2013; Danilov et al., 2015; Strong et al., 2008; Wan et al., 2013). Only one study in *C. elegans* showed the non-beneficial effects of aspirin for extending the lifespan. The failure might probably be due to the use of glp-1 mutant *C. elegans* as the worm model in this study (Huang et al., 2013). It is thought that GLP-1, a master regulator of germline development and longevity, is essential for the effects of aspirin on metabolism and lifespan extension in *C. elegans*. Therefore, the disruption of GLP-1 function will highly affect the effect of aspirin (Kenyon, 2010). In *C. elegans* studies, lifespan extending effect of aspirin can be explained by the activation of ampk and DAF-16/FOXO signaling pathway and oxidant stress prevention (Ayyadevar et al., 2013; Wan et al., 2013). Moreover, aspirin could downregulate Pkh2-ypk1-lem3-tat2 pathway in *drosophila* and act as an anti-inflammation compound in mice (Strong et al., 2008; Danilov et al., 2015). However, in healthy older adult population, aspirin failed to minimize mortality and morbidity and might have raised the risk of bleeding in such individuals (McNeil et al., 2018b; 2018a). This evidence showed the translational challenge of the use of aspirin in aging humans.

Studies on ACE-I have yielded conflicting results regarding its ability to extend lifespan, whereas all studies on ARB have shown no significant impact on prolonging lifespan. Candesartan failed to prolong the lifespan in UM-HET3 mice (Harrison et al., 2021). A study showed captopril extended lifespan in the dose at 2.5 mM (preferable dose) and 3.2 mM in *C. elegans* (Kumar et al., 2016). Another study demonstrated that captopril has a lifespan-extending effect at doses of 1.6, 2.5 (preferred dose), and 3.8 mM. However, this study revealed the toxicity of captopril in a dose of 7.6 mM. It might be because the drug dose in *C. elegans* should be lower than 3.8 mM (Egan et al., 2023). In the rodent models, captopril extended lifespan in female UM-HET3 mice (Strong et al., 2022). However, ramipril failed to extend the lifespan but was able to extend the lifespan when combined with simvastatin in C3B6F1 mice (Spindler et al., 2016). A detailed explanation will be given in the next section. Meanwhile, enalapril increased lifespan in Wistar rats by reducing leptin levels and ACE activity and enhancing the genes that involved lipid storage and antioxidant properties (Santos et al., 2009). A detailed explanation of these discrepancies in results was not shown in those studies but the different use of model organisms might explain the rationale explanation of these data.

The diuretic drugs, such as metolazone and acetazolamide extended the lifespan of *C. elegans* and Klotho hypomorphic (kl/kl) mice, respectively. Metolazone upregulates mitochondrial chaperone and activates mitochondrial unfolded protein response (UPRmt) to extend lifespan in worms, while acetazolamide inhibits osteoinductive signaling, ameliorates calcification markers, and reduces aldosterone and ADH levels in kl/kl mice (Ito et al., 2021; Leibrok al., 2015). Even though the lifespan was greatly increased by nearly 201% after acetazolamide treatment in kl/kl mice, the clinical translation into humans of this drug remains challenging since acetazolamide is widely applied as an eye drop for glaucoma treatment (Lusthaus and Goldberg, 2019). Hydralazine extended the lifespan in *C. elegans* in the optimal dose of 100 µM by activating SIRT1/SIR-2.1 and NRF2/SKN-1 signaling pathway, thus maintaining the mitochondrial function (Dehghan et al., 2017; 2019).

Anti-hypertensive medicines, such as beta-blockers (metoprolol and nebivolol) and verapamil were analyzed in this study. Sympathetic overdrive and overactivity in beta adrenergic receptors were found in aging organisms and led to age-associated cardiac failure (Lakatta, 1993; Swynghedauw et al., 1995). Metoprolol and nebivolol extended lifespan in both *drosophila* and mice by decreasing G proteins stimulation and reducing PKA activity in the heart after beta adrenergic receptor blockade. These drugs may also reduce tumor mass in mice (Spindler et al., 2013). Verapamil at the dose of 100 and 400 µM also increased the lifespan in *C. elegans* by reducing the calcineurin gene and enhancing LGG-1/LC3 expression level as the autophagy genes (Liu W et al., 2020).

### 4.2 Dyslipidemia drugs

The lifespan-extending effect has been identified in statin. Simvastatin increased the lifespan in *drosophila*, with the most significant impact observed at a dose of 0.24 mM, while lower or higher doses did not show the same effect (Spindler et al., 2012). This study found that simvastatin decreased Ras protein isoprenylation and reduced growth factor receptor signaling pathways to prolong the lifespan in *drosophila*. Another study also demonstrated that lovastatin extended the lifespan of *C. elegans* in a dose-dependent manner by preventing the accumulation of aging pigment and inhibiting the Jun N-terminal Kinase (JNK-1) pathway (Andreas et al., 2020). Interestingly, the beneficial effect of simvastatin alone could not be translated into mice. However, when combined with ramipril, the two drugs were able to prolong the lifespan by inhibiting AT1R signaling-mediated NAD(P)H oxidase inactivation, thus decreasing oxidative stress following ramipril administration (Spindler et al., 2016). Concerns were raised about hypertriglyceridemia and hyperglycemia when combining simvastatin and ramipril due to potential unexpected effects similar to those seen with a 40% calorie restriction diet and rapamycin administration (Spindler et al., 2016).

Niacin extended the lifespan in *C. elegans* only under high dose (Yang et al., 2019) and in Zucker Fatty rats (Preuss et al., 2011). In the worms study, it is suggested that niacin may raise intracellular nicotinamide adenine dinucleotide (NAD+) levels and maintain sirtuin-saturating concentrations to prolong lifespan (Yang et al., 2019). Moreover, fibrate treatment, specifically fenofibrate, extends lifespan in a dose-dependent manner by activating NHR-49 (an orthologue of Peroxisome Proliferator-Activated Receptor Alpha (PPAR-α) in mammals) to induce mitohormesis in *C. elegans* (Yang et al., 2019). We identified the conflicting data of lifespan-extending effect of omega-3 in *drosophila* and mice. Omega-3 may increase lifespan in *drosophila* males by increasing antioxidant enzymes and maintaining mitochondrial metabolism (Champigny et al., 2018). In contrast, this compound has rather shortened the lifespan in mice. The mechanism is not fully understood but it is speculated that the anticoagulant effects of omega-3 may induce bleeding risk and omega-3 can suppress CD8^+^ activation, thus inducing tumor progression in mice (Spindler et al., 2014; Champigny et al., 2018).

### 4.3 Anti-diabetic drugs

Metformin has been extensively studied in aging, including in lifespan studies. A total 15 metformin lifespan studies have been summarized in this review, including eight worm studies, two *drosophila* studies, four mice studies, and one rat study (Onken et al., 2022). Interestingly, the lifespan-extending effect of metformin is diverse among species. In worms, the beneficial effect of metformin was found when given at 0 days at the L4 larvae stage even though the therapeutic dose varied among studies (Onken et al., 2010; Cabreiro et al., 2013; Chen et al., 2017; Xiao et al., 2022; Cedillo et al., 2023). Metformin increased lifespan when administered at doses of 10, 25, and 50 mM starting at the L4 larvae stage from day 1, and at a dose of 50 mM from day 4. Moreover, the shortening of lifespan was detected when the metformin started at 10 days at the L4 larvae stage. This is probably due to the mitochondrial dysfunction caused by metformin toxicity at this stage (Espada et al., 2020). Another peculiar finding investigated by De Haes et al., 2014 that this study showed the lifespan-extending effect could happen when treating *C. elegans* with metformin 50 mM at the L1 stage and the adult phase only (De Haes et al., 2014). On the other hand, the advantageous effects of metformin on lifespan was found limited to *C. elegans* but not *C. briggsae* or *C. tropicalis* suggesting that metformin works in the specific target organism (Onken et al., 2022).

In contrast with the metformin effect in worms, metformin rather reduced lifespan in *drosophila* (Slack et al., 2012; Abrat et al., 2018). Metformin at the dose of 100 mM in male and more than 25 mM in females may induce the shortening of lifespan in *drosophila*. These phenomena could be caused by the starvation-like phenotype and intestinal fluid imbalance due to overactivity AMPK signaling induced by metformin intoxication (Slack et al., 2012). Similarly, another study also found this unexpected phenotype although it speculated that the starch diet used in this study might disrupt metabolic homeostasis in *drosophila* (Abrat et al., 2018). Altogether, these data showed the unexpected effects of metformin in this organism.

Metformin has no beneficial effect on lifespan in F344 male rats and rather decreases the body weight of this rat (Smith et al., 2010). Moreover, the gender-specific lifespan effect of metformin has been observed in mouse models. Metformin extended lifespan in male 129/SV (100 mg/kgBW/day) and C57BL/6 mice (0.1% w/w). Renal toxicity occurred in C57BL/6 mice when treated with the dose at 1%w/w (Montalvo et al., 2013; Anisimov et al., 2015). Moreover, metformin decreased lifespan at 129/SV and C57BL/6 female mice, even when administered at the similar dose in the male study (100 mg/kgBW/day) (Montalvo et al., 2013; Zhu et al., 2021). The administration of metformin in female mice may result in an elevated level of cardiac stress indices, such as Myh7/Myh6, Nppa, and Nppb, which can account this event (Zhu et al., 2021). Another fact revealed that a combination of metformin 1,000 ppm and rapamycin 14 ppm prolonged lifespan in both male and female UM-HET3 mice even though the metformin 1,000 ppm was not sufficient to promote this phenotype. As mentioned previously, this mechanism can be interpreted by the hypothesis that metformin improves glucose homeostasis by enhancing the insulin sensitivity that is perturbated by rapamycin (Strong et al., 2016).

Among all anti-diabetic drugs, acarbose has been identified as the most consistent compound for extending lifespan in rodents, but it failed to prolong the lifespan of various types of worms (Banse et al., 2023). Studies showed that acarbose at a dosage of 1,000 ppm had positive effects on C3D2F1/J or CByB6F1/J mice when treated from 8 months old, and on UM-HET3 mice when treated from 4, 8, 9 months old, respectively (Harrison et al., 2014; 2019; Strong et al., 2016; Smith et al., 2019). This lifespan-extending effect might be due to the change in microbiome composition and fecal Short-Chain Fatty Acids production. The increase of FGF21 and reduction of IGF-1 plasma levels may also be involved as the molecular mechanism of lifespan-extension phenotype in acarbose (Harrison et al., 2014; 2019; Smith et al., 2019). When started at 16 months old, acarbose at the dose of 1,000 ppm extended its lifespan in male mice only. However, when combined with rapamycin at the dose of 14.7 ppm, acarbose was able to extend the lifespan of both genders, even when the initial treatment started at 16 months old (Strong et al., 2022). This synergistic effect might be explained by the insulin-sensitizing effect of acarbose could neutralize the hyperglycemia condition caused by rapamycin. These studies collectively suggest the potential role of translating acarbose for extending lifespan in aging humans.

Thiazolidinediones (TZD), such as pioglitazone and rosiglitazone, have been reviewed in this study. Pioglitazone extended lifespan at concentrations of 0.1 and 0.5 mM but failed when given less than 0.1 or at 2 mM in *C. elegans* (Jia et al., 2022; Onken et al., 2022). This effect is due to the activation of DAF-16/FOXO and SKN-1/NRF2 Signaling Pathways while inhibiting insulin/insulin-like signaling (IIS) and reproductive signaling pathways, as well as the activation of dietary restriction-related pathway (Jia et al., 2022). Unfortunately, no further studies in larger organisms than worms have been identified in TZD. Similar to TZD, the lifespan study of sulphonylureas (chlorpropamide, glibenclamide, glimepiride, and glipizide) has been limited to *C. elegans* only (Mao et al., 2022; Onken et al., 2022). Our systematic review showed that all sulphonylureas, except for glipizide, increased lifespan at different doses. The increases of the mitochondrial electrical potential and SDH activity in Complex II, and mitochondrial reactive oxygen species (mtROS) play the molecular mechanism of this lifespan-extending effect (Mao et al., 2022). In addition, nateglinide was unlikely to shorten the lifespan in *C. elegans* and *C. tropicalis* although the mechanism remains unknown (Onken et al., 2022).

Sodium-glucose transport Protein 2 (SGLT2) Inhibitors showed different results between worm and mice studies (Miller et al., 2020; Onken et al., 2022). When administered at a maximum dose of 100 μM, dapagliflozin did not increase the lifespan of various types of worms (Onken et al., 2022). However, when given at a dose of 180 ppm to UM-HET3 male mice starting at 7 months old, canagliflozin extended their lifespan, but not that of the female mice. The valid mechanism has not been established yet, but it speculated that canagliflozin enhances fatty acids and ketones metabolism, suppresses the TORC1 signaling pathway, and increases AMPK activity in liver tissue (Miller et al., 2020). The effect of lifespan extension of Dipeptidyl Peptidase 4 (DPP 4) Inhibitors such as sitagliptin and linagliptin have been investigated in various types of worms and Klotho^−/−^ mice, respectively (Hasegawa et al., 2017; Onken et al., 2022). The analysis of sitagliptin at a maximal dose of 100 µM in worms found that lifespan extension phenotype has only happened in *C. elegans* and *C. tropicalis*, but not in *C. briggsae* (Onken et al., 2022). On the other hand, linagliptin extended lifespan in Klotho^−/−^ mice model by enhancing phosphorylation activities of Akt, eNOS, and CREB in the brain. However, the treatment of linagliptin in this model induced hyperglycemia status and increased body weight (Hasegawa et al., 2017). Consistent with our findings, another review of the potential of gerotherapeutic drugs revealed that SGLT-2 exhibits superior efficacy in extending preclinical lifespan compared to metformin (Kulkarni et al., 2022).

### 4.4 Lesson from animal aging models

According to our findings, five distinct species were utilized in the aging drug repositioning study. Worms, flies, mice, and rats are the most frequently utilized in aging trials, respectively. These species are utilized on account of their properties in easy handling, short generation times, availability of standardized strain, and high quality in genomic and transcriptomic sequencing data (de Magalhães, 2021; Holtze et al., 2021).

Roundworms (*C. elegans*) are predominantly employed in aging trials because of their simple cultivation and brief life cycle and life span (two to 3 weeks). Furthermore, 50% of *C. elegans* genes are present in the human genome (Taormina et al., 2019). *D. melanogaster*, a higher animal frequently used in lifespan studies, possesses four pairs of chromosomes and functional orthologues for sixty percent of the genes implicated in human diseases. This characteristic renders the fruit fly a more suitable subject for lifespan studies (Taormina et al., 2019). Additionally, our research uncovered one article that utilized silkworm (*Bombyx mori*) as an animal model (Song et al., 2019). An additional noteworthy characteristic of this model is its profusion of three to six larval instars, in contrast to three larval instars in *D. melanogaster*. This increased the plasticity of lifespan extension (Song et al., 2017).

Mice contain almost 99% human orthologue genes, making them one of the most appropriate for human models. However, their studies are more complex and challenging because they are higher animals. Additionally, higher animals possess advantageous system organs, including but not limited to the musculoskeletal apparatus, endocrine system, and immune system, which can be modified to target drugs of action selectively (Taormina et al., 2019). Rats, similar to mice, are a fascinating species to investigate in the context of lifespan. Rats are more prone to developing cardiovascular and renal diseases, rendering them more disease-prone in comparison to mice. However, rats have a lower cancer incidence (74%–88% compared with 83%–95% in mice). These characteristics indicate that rats have a narrower margin for the prevalence of cardiovascular, cancer, and renal diseases in humans (Carter et al., 2020). However, in metabolic-focused research, such as insulin resistance, mice are preferable to rats due to their extensive use and the well-established development of transgenic mice for insulin resistance (Berglund et al., 2008). Thus, research on the aging of mice and rats should be considered more representative of the human condition.

Interpretation bias can arise from species variation caused by specific characteristics of the species being studied, leading to inaccurate generalizations (Holtze et al., 2021). For instance, sirtuin extends the lifespan of yeast through Sir2-mediated mechanisms (Kaeberlein et al., 1999), but this effects not well replicated in higher animal models (Park et al., 2013). Our study further supports a distinct attribute of species by revealing that metformin can prolong the lifespan of *C. elegans* while diminishing it in *C. tropicalis* (Park et al., 2013). Hypothetically, this distinction occurred due to the distinction between the epithelial boundary and the *Caenorhabditis cuticle*, which distinguishes the ability of metformin to penetrate *Caenorhabditis* cells (Holden-Dye and Walker, 2014; Onken et al., 2022).

Utilizing exclusively normal strains of animals may occasionally give rise to an additional issue. Normal animal strains typically result in restricted genetic diversity, which may not consistently apply to clinical applications in the considerably more heterogeneous human population (de Magalhães, 2014). As an illustration, C57BL/6 mice, which have been utilized in 70% of published animal studies, exhibit a higher incidence of lymphoma and increased vulnerability to metabolic dysregulation (Ward, 2006; Mitchell et al., 2015). Information derived from a solitary inbred strain might lack generalizability to the entire species. Moreover, the genetic uniformity that ensues from the breeding of strains is not indicative of the human population (Mitchell et al., 2015). As a result, genetically modified animals can occasionally serve to advance our understanding of genetic diversity.

Our systematic review also incorporates genetically modified mice that demonstrate premature aging; however, we do not incorporate animals with disease models. Several Klotho mouse studies were included in this systematic review (Leibrock et al., 2016; Hasegawa et al., 2017). Over 2 decades ago, Klotho was implemented as a gene modification in an aging model. The phenotype of these mice klotho modified includes frailty, vascular calcification, cardiovascular disease, and multiple organ degeneration (Kuro-o et al., 1997). Furthermore, recent studies have demonstrated that klotho serum levels play a role in the aging process and physical function of humans (Arroyo et al., 2023). It is noteworthy that human klotho serum levels exhibited a U-shaped curve. In participants with low Klotho serum, the phenotypic age acceleration decreased significantly with increasing serum Klotho, whereas it increased in participants with high Klotho serum (Li et al., 2023). However, the mechanism of this phenomenon is still unclear.

Gender differences in the lifespan-extending effect likely happened in some of the studies. Most drugs have been tested and proven effective in male mice but not in female mice. For instance, anti-diabetic drugs are more effective in male than female mice. The mechanism behind this phenomenon is not completely understood, but research suggests that certain drugs may interact with sex hormones and impact the reproductive organs of a particular gender (Garratt, 2020). Another concern that needs to be addressed is determining the optimal timing for administering longevity compounds to animal models. Additional research is needed to clarify the gender- and time-specific impacts of gerotherapeutics.

Selecting the correct therapeutic dosage is crucial to avoid a false negative outcome or unforeseen intoxication. It is advisable to utilize the dosage specified in previous literature or to modify the therapeutic dosage based on a human study. Pharmacokinetic variations among organisms should be taken into account to establish the correct dosage, especially for long-term use in lifespan studies (Spindler, 2012).

Various side effects, including severe ones, have been identified in this study, such as the bleeding risk and malignancy phenotype that can be found in omega-3 and the risk of renal failure or mitochondrial dysfunction that might occur in metformin treatment (Montalvo et al., 2013; Spindler et al., 2014; Espada et al., 2020; Zhu et al., 2021). The interplay between drugs and lifestyle variables is complex and needs careful deliberation. Cardiometabolic and antidiabetic medications offer significant benefits in clinical settings. Their integration with exercise and dietary therapies may yield diverse results. Although statin is generally beneficial for reducing cholesterol levels and minimizing the risk of cardiovascular events, this medicine has been connected with muscle-related adverse effects, such as myalgia, which can hinder physical performance during exercise (Parker et al., 2013). Of note, statin might potentially diminish the beneficial impacts of exercise on muscle adaptability and mitochondrial function. While Beta-blocker provides cardiovascular protection by reducing heart rate, their effect on exercise tolerance and performance is frequently detrimental, which may discourage physical activity in patients. Metformin, an important therapy for type 2 diabetes, has undergone substantial research to explore its potential as an anti-aging medication. However, metformin may potentially impede the beneficial effect of aerobic exercise on cardiorespiratory fitness and insulin sensitivity by reducing the mitochondrial adaptations to exercise (Konopka et al., 2019). Additionally, diets rich in fiber may hinder the absorption of certain medicines, including statin and beta blocker, thus decreasing their effectiveness as well (Jenkins et al., 2000).

### 4.5 Future direction in repurposing cardiometabolic drugs for aging

Our systematic review of animal study results indicates that several drugs have the potential to enhance lifespan. However, as this is solely an animal study, its impact may vary in human studies. Moreover, certain animals can exhibit a better representation of human characteristics compared to other animals. Overall, rats and mice exhibit a stronger weight of evidence compared to *Drosophila*, whereas *C. elegans* demonstrates the lowest weight of evidence based on gene orthologue (Holtze et al., 2021). Hence, it is important to carry out human clinical trials on this subject. Further discussion will focus on the latest developments in human clinical trials for cardiometabolic drugs that are related to improving both healthspan and lifespan.

The anti-aging effects of metformin have been the subject of extensive animal and human testing as part of the TAME (Targeting Aging with Metformin) initiative (Barzilai et al., 2016). Three thousand nondiabetic adults aged 65 to 80 will participate in the TAME clinical trial, a 6-year double-blind, randomized, placebo-controlled study. Metformin slow-release 1,500 mg will be administered (AFAR, 2023). IL-6, TNFα-receptor I or II, CRP, GDF15, insulin, IGF1, cystatin C, NT-proBNP, and hemoglobin A1c biomarkers will be utilized in this investigation, as they have been demonstrated to be the most accurate predictors of numerous biological aging processes (Justice et al., 2018). Hopefully, these biomarkers can also be implemented in future human aging research.

The Antedecendent Metabolic Health and Metformin (ANTHEM) Aging Study is an additional noteworthy clinical trial on metformin and aging. Hundreds of participants in a shorter period will be enrolled so that results can be anticipated before TAME. It will assess changes in insulin sensitivity and mitochondrial transport system by skeletal muscle biopsy (Kumari et al., 2022). We also noted the finished Metformin in Longevity Study (MILES), which demonstrated that after 6 weeks of administration to older adults, metformin regulated numerous metabolic and nonmetabolic pathways in skeletal muscle and subcutaneous adipose tissue. Metformin exerted its effects not solely on metabolic genes and pathways but also on DNA repair genes in muscle and mitochondrial genes in adipose tissue (Kulkarni et al., 2018).

However, our systematic review of animal trials indicates that metformin does not consistently extend life expectancy. This is the first systematic review to examine animals without disease induction. Other meta-analyses conducted on animal trials indicate that the efficacy of metformin is limited to *C. elegans* when administered early in life, while its effects vary among the other model organisms (Parish and Swindell, 2022). This raises further inquiries, such as whether the TAME and ANTHEM study will yield favorable outcomes considering its heterogeneous impact on the other animal species.

We also notice an ongoing clinical trial on metformin in patients with HIV (EUCTR 2021-003299-15-ES). An individual afflicted with HIV is subjected to a multitude of stressors, including the virus, antiretroviral medications, and substances misused, all of which have the potential to trigger premature cellular senescence (Cohen and Torres, 2017). Therefore, HIV-positive individuals are incorporated into this clinical trial registry review as they exhibit early cellular aging. This study employs the potentially effective Epigenetic Age Acceleration (EAA) for its primary outcome, which is intended to predict lifespan (Joyce et al., 2021).

Three metformin registries appear incomplete for various reasons, including participant assignment difficulties. Additionally, we identified studies with dropout rates exceeding 20%, which diminished the conclusions’ reliability. Hopefully, future clinical trials involving older individuals will incorporate improved recruitment and retention strategies and more efficient planning and execution. One of the solutions is illustrated in the cited source (Chaudhari et al., 2020).

Besides metformin, omega-3 fatty acids have been shown to reduce telomere attrition via antioxidant effect, decreased proinflammatory markers, and direct action on telomeres based on *in vitro* and *in vivo* studies; these effects have promising implications for health and longevity (Ogłuszka et al., 2022). In addition, levels of long-chain omega-3 fatty acids were found to be inversely associated with mortality in the Framingham Heart Study (Harris et al., 2018).

Results from three studies concerning omega-3 in healthy subjects have been published. Although omega-3 fatty acids have no discernible impact on immunosenescence pathway (Swanson et al., 2018), they have been found to influence cognitive function in healthy humans positively (Külzow et al., 2016; Fairbairn et al., 2020). While the precise mechanism by which omega-3 fatty acids influence cognitive function remains unknown, they do regulate the expression of genes encoding enzymes involved in homocysteine metabolism, amino acids that correlate to neuronal senescence (Huang et al., 2012; Tawfik et al., 2021).

Omega-3 dose is also a challenge in the aging trial. No fixed EPA and DHA dose combination proves to increase lifespan. A systematic review also showed that no fixed dose has been established in the cognitive area. It only shows that significantly altered neurophysiological function or brain morphology can be achieved with prolonged omega-3 administration (Dighriri et al., 2022). One study also uses the combination of folic acid, phosphatidylserine, and *Gingko biloba*, thereby augmenting the treatment effect bias (Külzow et al., 2016). In conclusion, omega-3 fatty acids may extend the cognitive healthspan of healthy individuals. However, additional research employing rigorous methodologies is required to determine whether it extends lifespan.

Two registries (NCT02865499 and NCT02953093) are evaluating the fecal microbiome of healthy older individuals after acarbose treatment (8 and 10 weeks). One of these registries also analyses gene expression in abdominal adipose tissue and muscle tissue. None of the results has been published yet. Animal studies indicate that acarbose is hypothesized to extend lifespan through modifications to the gut microbiome and an increase in short-chain fatty acids (SCFAs), including propionate (Smith et al., 2019). As a result, the gut microbiome serves as a reliable biomarker for the effect of acarbose on longevity. Nevertheless, it is desirable that future research incorporates larger sample sizes and more prolonged acarbose administration periods to interpret the effect size more accurately.

Statins for Extension of Disability-Free Survival and Primary Prevention of Cardiovascular Events Among Older People (STAREE) is also an intriguing study in the aging field. This investigation comprises the STAREE-HEART and STAREE-MIND substudies (Harding et al., 2023; Zoungas et al., 2023). STAREE-HEART will examine the incidence of atrial fibrillation and global longitudinal strain (GLS) in healthy older adults taking 20 mg of atorvastatin daily over a 3-year follow-up period. Following a 4-year follow-up period, STAREE-MIND will assess brain aging parameters and investigate the correlation between changes in brain imaging and cognitive impairment. Hopefully, the findings of this research will shed light on the impact of atorvastatin on healthy subjects’ cardiovascular and neurological aging.

Several included articles incorporated cardiometabolic drugs that were discontinued in human application due to safety concerns (Brandstädt et al., 2013; Cabreiro et al., 2013; Mao et al., 2022; Cedillo et al., 2023). These are bezafibrate (hepatotoxicity), tolbutamide (cardiovascular mortality), chlorpropamide (hypoglycemia), and phenformin (lactic acidosis). Animal lifespan research aims to identify the most effective medication for extending human health and lifespan. So, we encourage further lifespan research to avoid the use of discontinued drugs, particularly when safety concerns arise.

### 4.6 Limitations

Our study has several limitations. First, our search was limited to drugs that extend lifespan, not healthspan. As a result, articles that do not provide follow-up of the animal until death were excluded. Second, we excluded animals with disease models, as they are inappropriate for our PICO. These two limitations may result in less comprehensive cardiometabolic drug mechanisms in aging. Third, our search criteria exclusively included drugs that have received approval from the FDA, which has been established for their efficacy and safety in treating cardiometabolic disease. Hopefully, it can also be safe as future potential human lifespan-extending drugs. Fourth, most of the medications in rodents studies were given orally. However, the majority of these studies did not explicitly mention whether the medications were administered orally or mixed with chow. Additionally, drug concentrations for *C. elegans* and *Drosophila* studies are commonly used in the millimolar (mM) range format. This approach is in agreement with the established protocols in aging research when evaluating pharmacological interventions in these model organisms. Lastly, regarding clinical trials, our search criteria in ICTRP are limited to the keyword “aging”. Some clinical trial registries may repurpose cardiometabolic drugs for degenerative conditions but not use aging as their keyword. It is possible that such research may not be included in this systematic review.

## 5 Conclusion

Metformin, omega-3 fatty acid, acarbose, and atorvastatin are currently cardiometabolic drugs repurposed to target aging in clinical trials. Our systematic review of animal trials identified several additional cardiometabolic drugs that could potentially extend life expectancy. We strongly advise other researchers to initiate clinical trials of these drugs in the context of aging, given the significant concern that this will become in the coming years. Additional animal experiments utilizing wild-strain animals to evaluate the effects of gerotherapeutics are also recommended.

## Data Availability

The original contributions presented in the study are included in the article/Supplementary Material, further inquiries can be directed to the corresponding authors.
