# Supplementary material for: Repurposing effect of cardiovascular-metabolic drug to increase lifespan: a systematic review of animal studies and current clinical trial progress
Source: Front Pharmacol. 2024 Jun 20;15:1373458. doi: 10.3389/fphar.2024.1373458 (PMC11223003; doi:10.3389/fphar.2024.1373458)
Supplement: Supplementary file 1 [file Table1.DOCX]

Supplementary Table 1. Search strategies (performed in September 2023)

| **Database** | **Keywords** |
| --- | --- |
| Pubmed | (cardiometabolic drugs[Title/Abstract]) AND (lifespan[Title/Abstract]) AND (animal[Filter]) |
| Embase | 'cardiometabolic drugs':ab AND 'lifespan':ab AND [animals]/lim |
| Web of Science | (AB=(cardiometabolic drugs)) AND AB=(lifespan) Document Types: Articles |
| Scopus | TITLE-ABS-KEY ( “cardiometabolic drugs” AND lifespan ) AND ( LIMIT-TO ( EXACTKEYWORD , "Nonhuman" ) ) AND ( LIMIT-TO ( DOCTYPE , "ar" ) ) |

Each cardiometabolic drug was searched individually based on these AHA (American Heart Association) and ADA (American Diabetes Association) lists:

Heart medications: <https://www.heart.org/en/health-topics/heart-attack/treatment-of-a-heart-attack/cardiac-medications>

Diabetes medications: <https://diabetes.org/healthy-living/medication-treatments/oral-other-injectable-diabetes-medications>

Cholesterol medications: <https://www.heart.org/en/health-topics/cholesterol/prevention-and-treatment-of-high-cholesterol-hyperlipidemia/cholesterol-medications>

Supplementary Table 2. Total number of articles found for each cardiometabolic drug

| **Drug Name** | **Pubmed (Medline)** | **Embase** | **Web of Science** | **Scopus** |
| --- | --- | --- | --- | --- |
| **Cardiovascular Drugs** | | | | |
| **Anticoagulants** | | | | |
| Apixaban | 0 | 0 | 1 | 0 |
| Dabigatran | 0 | 0 | 0 | 1 |
| Edoxaban | 0 | 0 | 0 | 0 |
| Heparin | 21 | 22 | 46 | 24 |
| Rivaroxaban | 0 | 0 | 0 | 1 |
| Warfarin | 1 | 1 | 10 | 3 |
| **Antiplatelet Agents and Dual Antiplatelet Therapy (DAPT)** | | | | |
| Aspirin | 24 | 28 | 28 | 41 |
| Clopidogrel | 0 | 0 | 2 | 4 |
| Dipyridamole | 0 | 0 | 0 | 0 |
| Prasugrel | 0 | 0 | 0 | 2 |
| Ticagrelor | 0 | 0 | 0 | 1 |
| **Angiotensin-Converting Enzyme (ACE) Inhibitors** | | | | |
| Benazepril | 1 | 1 | 1 | 2 |
| Captopril | 10 | 11 | 7 | 8 |
| Enalapril | 3 | 4 | 1 | 9 |
| Fosinopril | 0 | 0 | 0 | 1 |
| Lisinopril | 2 | 0 | 1 | 3 |
| Moexipril | 0 | 0 | 0 | 0 |
| Perindopril | 2 | 2 | 1 | 5 |
| Quinapril | 0 | 0 | 0 | 1 |
| Ramipril | 9 | 15 | 11 | 12 |
| Trandolapril | 1 | 1 | 1 | 1 |
| **Angiotensin II Receptor Blockers (or Inhibitors)** | | | | |
| Azilsartan | 0 | 0 | 0 | 0 |
| Candesartan | 7 | 9 | 7 | 6 |
| Eprosartan | 0 | 0 | 0 | 0 |
| Irbesartan | 0 | 0 | 0 | 1 |
| Losartan | 11 | 16 | 10 | 16 |
| Olmesartan | 2 | 2 | 2 | 2 |
| Telmisartan | 1 | 1 | 1 | 1 |
| Valsartan | 2 | 2 | 2 | 2 |
| **Angiotensin Receptor-Neprilysin Inhibitors (ARNIs)** | | | | |
| Sacubitril/valsartan | 0 | 0 | 0 | 0 |
| **Beta Blockers** | | | | |
| Acebutolol | 0 | 0 | 0 | 0 |
| Atenolol | 1 | 2 | 3 | 6 |
| Betaxolol | 0 | 0 | 0 | 0 |
| Bisoprolol | 0 | 5 | 2 | 0 |
| Metoprolol | 1 | 0 | 1 | 4 |
| Nadolol | 0 | 0 | 0 | 1 |
| Propranolol | 0 | 7 | 6 | 12 |
| Sotalol | 5 | 1 | 1 | 2 |
| **Combined Alpha and Beta-Blockers** | | | | |
| Carvedilol | 0 | 0 | 0 | 1 |
| Labetalol | 0 | 0 | 0 | 0 |
| **Calcium Channel Blockers** | | | | |
| Amlodipine | 2 | 2 | 4 | 8 |
| Diltiazem | 0 | 0 | 0 | 1 |
| Felodipine | 0 | 0 | 0 | 0 |
| Nifedipine | 1 | 4 | 1 | 4 |
| Nimodipine | 3 | 3 | 2 | 3 |
| Nisoldipine | 0 | 0 | 0 | 0 |
| Verapamil | 7 | 8 | 4 | 12 |
| **Digitalis Preparations** | | | | |
| Digoxin | 2 | 4 | 2 | 8 |
| **Diuretics** | | | | |
| Acetazolamide | 3 | 3 | 2 | 3 |
| Amiloride | 4 | 8 | 5 | 4 |
| Bumetanide | 2 | 1 | 1 | 4 |
| Chlorothiazide | 2 | 1 | 1 | 0 |
| Chlorthalidone | 0 | 0 | 0 | 0 |
| Furosemide | 1 | 1 | 1 | 7 |
| Hydrochlorothiazide | 2 | 0 | 0 | 2 |
| Indapamide | 2 | 1 | 0 | 1 |
| Metalozone | 0 | 0 | 1 | 0 |
| Spironolactone | 3 | 6 | 4 | 9 |
| Torsemide | 0 | 0 | 0 | 0 |
| **Vasodilators** | | | | |
| Isosorbide dinitrate | 0 | 0 | 1 | 0 |
| Isosorbide mononitrate | 0 | 0 | 0 | 1 |
| Hydralazine | 11 | 7 | 7 | 9 |
| Nitroglycerin | 0 | 1 | 0 | 1 |
| Minoxidil | 0 | 1 | 0 | 0 |
| **Dyslipidemia Drugs** | | | | |
| **Statin** | | | | |
| Atorvastatin | 2 | 2 | 3 | 13 |
| Rosuvastatin | 1 | 0 | 0 | 5 |
| Fluvastatin | 0 | 0 | 1 | 1 |
| Simvastatin | 4 | 3 | 4 | 10 |
| Pravastatin | 2 | 2 | 1 | 4 |
| Lovastatin | 1 | 5 | 1 | 2 |
| **Bile Acid Sequestrant** | | | | |
| Cholestyramine | 0 | 0 | 0 | 0 |
| Colestipol | 0 | 0 | 0 | 0 |
| Colesevelam | 0 | 0 | 0 | 0 |
| Ezetimibe | 1 | 0 | 1 | 1 |
| **PCSK9 Inhibitor** | | | | |
| Alirocumab | 0 | 0 | 0 | 0 |
| Evolocumab | 0 | 0 | 0 | 0 |
| **Adenosine Triphosphate Citrate Lyase Inhibitor** | | | | |
| Bempedoic Acid | 0 | 0 | 0 | 0 |
| **Fibrates** | | | | |
| Gemfibrozil | 0 | 0 | 0 | 1 |
| Fenofibrate | 4 | 2 | 2 | 6 |
| Clofibrate | 3 | 3 | 2 | 4 |
| **Niacin** | | | | |
| Niacin | 7 | 6 | 4 | 10 |
| Omega 3 | | | | |
| Omega 3 Fatty Acid Ethyl Esters | 0 | 0 | 0 | 3 |
| Marine-derived Omega 3 Polyunsaturated Fatty Acid (PUFA) | 0 | 2 | 1 | 2 |
| **Diabetic Drugs** | | | | |
| **Metformin** | | | | |
| Metformin | 111 | 111 | 105 | 131 |
| **DPP-4 inhibitors** | | | | |
| Alogliptin | 2 | 1 | 1 | 1 |
| Linagliptin | 1 | 3 | 1 | 1 |
| Saxagliptin | 0 | 0 | 0 | 0 |
| Sitagliptin | 1 | 0 | 0 | 1 |
| **GLP-1 agonists** | | | | |
| Dulaglutide | 0 | 0 | 0 | 0 |
| Exenatide | 1 | 1 | 1 | 4 |
| Liraglutide | 3 | 4 | 5 | 4 |
| Lixisenatide | 0 | 0 | 0 | 0 |
| Semaglutide | 1 | 1 | 3 | 0 |
| **SGLT2 inhibitors** | | | | |
| Bexagliflozin | 0 | 0 | 0 | 0 |
| Canagliflozin | 5 | 6 | 5 | 7 |
| Dapagliflozin | 2 | 2 | 3 | 3 |
| Empagliflozin | 2 | 2 | 2 | 4 |
| **Sulfonylureas** | | | | |
| Glimepiride | 1 | 3 | 3 | 4 |
| Glipizide | 0 | 0 | 0 | 5 |
| Glyburide | 0 | 0 | 0 | 2 |
| Glibenclamide | 2 | 2 | 2 | 8 |
| **Thiazolidinediones** | | | | |
| Rosiglitazone | 7 | 7 | 5 | 13 |
| Pioglitazone | 7 | 11 | 7 | 11 |
| **Alpha-glucosidase inhibitors** | | | | |
| Acarbose | 21 | 37 | 22 | 24 |
| Miglitol | 0 | 0 | 0 | 0 |
| **Bile acid sequestrant** | | | | |
| Colesevelam | 0 | 0 | 0 | 0 |
| **Dopamine-2 agonist** | | | | |
| Bromocriptine | 6 | 5 | 3 | 5 |
| **Meglitinides** | | | | |
| Nateglinide | 0 | 0 | 0 | 1 |
| Repaglinide | 0 | 0 | 0 | 0 |
| **Total article found** | 347 | 402 | 369 | 541 |

Supplementary Table 3. Risk of bias in animal trial studies

| **No.** | **First author, year** | **Selection bias** | | | **Performance bias** | | **Detection bias** | | **Attrition bias** | **Reporting bias** | **Other** |
| --- | --- | --- | --- | --- | --- | --- | --- | --- | --- | --- | --- |
|  |  | **Sequence generation** | **Baseline characteristics** | **Allocation concealment** | **Random housing** | **Blinding** | **Random outcome assessment** | **Blinding** | **Incomplete outcome data addressed** | **Free of selective outcome reporting** | **Other sources of bias** |
| 1 | (Spindler et al., 2016) | ? | + | ? | + | ? | - | + | ? | + | + |
| 2 | (Spindler et al., 2012) | ? | + | + | + | ? | ? | + | + | + | + |
| 3 | (Spindler et al., 2014) | ? | + | ? | + | ? | ? | + | + | + | + |
| 4 | (Spindler et al., 2013) | ? | + | ? | + | ? | - | + | + | + | + |
| 5 | (Smith et al., 2019) | ? | ? | ? | + | + | ? | + | + | + | + |
| 6 | (Smith et al., 2010) | ? | + | ? | + | ? | ? | + | + | + | + |
| 7 | (Song et al., 2019) | ? | + | ? | + | ? | ? | + | + | + | + |
| 8 | (Strong et al., 2016) | + | + | ? | + | ? | ? | + | + | + | + |
| 9 | (Harrison et al., 2014) | + | + | - | - | - | - | + | + | + | + |
| 10 | (Harrison et al., 2019) | + | + | ? | - | - | - | + | + | ? | ? |
| 11 | (Harrison et al., 2021) | + | + | ? | - | - | - | + | + | ? | ? |
| 12 | (Hasegawa et al., 2013) | - | + | - | - | - | - | + | ? | ? | ? |
| 13 | (Huang et al., 2017) | - | + | - | - | - | - | + | ? | ? | + |
| 14 | (Ito et al., 2021) | - | + | ? | - | - | - | + | ? | ? | - |
| 15 | (Andreas et al., 2020) | - | + | ? | + | + | + | + | ? | ? | ? |
| 16 | (Jia et al., 2022) | - | - | - | - | - | - | - | ? | ? | ? |
| 17 | (Santos et al., 2009) | - | + | - | + | - | - | - | + | ? | - |
| 18 | (Leibrok et al, 2015) | - | + | - | ? | - | - | + | ? | ? | ? |
| 19 | (Slack et al., 2012) | - | + | - | + | - | - | + | ? | + | ? |
| 20 | (Kumar et al., 2016) | - | + | - | ? | - | - | + | + | + | + |
| 21 | (Mao Z. et al., 2021) | - | + | - | - | - | - | + | ? | + | ? |
| 22 | (Montalvo et al., 2013) | - | + | - | ? | - | - | + | ? | + | ? |
| 23 | (Liu W. et al., 2020) | - | + | - | ? | - | ? | + | ? | ? | ? |
| 24 | (Dehghan et al., 2019) | - | + | - | - | - | - | + | + | + | + |
| 25 | (Dehghan et al., 2017) | - | + | - | - | - | - | + | + | + | + |
| 26 | (Egan et al., 2023) | - | + | - | - | - | ? | + | + | + | + |
| 27 | (Espada et al., 2020) | ? | + | ? | + | - | - | + | + | + | + |
| 28 | (Preuss et al., 2011) | - | + | - | ? | ? | ? | + | + | + | + |
| 29 | (Strong et al.,2008) | + | + | ? | + | + | + | + | + | + | + |
| 30 | (Strong et al., 2022) | + | + | ? | + | + | + | + | + | + | + |
| 31 | (Xiao et al., 2022) | - | + | - | - | - | - | + | + | + | + |
| 32 | (Xu et al., 2020) | - | + | - | ? | - | - | + | ? | ? | + |
| 33 | (Zhu et al., 2021) | - | + | - | - | - | - | + | ? | ? | + |
| 34 | (Wan et al., 2013) | - | + | - | + | + | - | + | ? | ? | ? |
| 35 | (Yang et al., 2019) | - | + | - | - | - | - | + | ? | ? | + |
| 36 | (Miller et al. 2020) | + | + | + | + | + | + | + | + | + | + |
| 37 | (Abrat et al., 2018) | - | + | - | - | - | - | + | ? | + | + |
| 38 | (Ayyadevara et al., 2013) | - | + | - | - | - | - | + | + | + | - |
| 39 | (Banse et al., 2023) | - | + | - | + | - | + | + | + | + | ? |
| 40 | (Anisimov et al., 2015) | - | + | - | - | - | + | + | + | + | - |
| 41 | (Brandstädt et al., 2013) | ? | + | - | - | - | + | + | + | + | ? |
| 42 | (Cabreiro et al., 2013) | - | + | - | - | - | + | + | + | + | ? |
| 43 | (Onken et al., 2010) | - | + | - | ? | ? | ? | + | + | + | + |
| 44 | (Champigny et al., 2018) | ? | + | - | ? | ? | ? | + | + | + | + |
| 45 | (Cedillo et al., 2023) | ? | ? | - | ? | + | ? | + | + | + | + |
| 46 | (Chen et al., 2017) | ? | + | - | ? | ? | ? | + | + | + | + |
| 47 | (Danilov et al., 2015) | ? | + | - | ? | ? | ? | + | + | + | + |
| 48 | (De Haes et al., 2014) | ? | + | - | ? | ? | ? | + | + | + | + |
| 49 | (Onken et al., 2021) | - | + | - | ? | ? | ? | + | + | + | + |

Supplementary Table 4. Result of animal trial studies

| **No** | **(First author, year)** | **Species strain** | **Genetically modified aging** | **Gender** | **Intervention (dose)** | **Age at treatment initiation** | **Total subjects** | **Lifespan** | **Molecular mechanism of lifespan increment** | **Cardiometabolic outcome** | **Musculoskeletal outcome** | **Others outcomes** |
| --- | --- | --- | --- | --- | --- | --- | --- | --- | --- | --- | --- | --- |
| 1 | (Spindler et al., 2016) | C3B6F1 mice | No | Male | Ramipril (5.0 mg/kgBW/day) | 12 months | Exp: 36 Con: 72 | NS | Several hypotheses were speculated:  - Simvastatin alters energy homeostasis and disposition. However, simvastatin blunted insulin sensitivity-induced new onset of type 2 diabetes, and ramipril increased serum triglyceride levels, mimicking hypertriglyceridemia and hypercholesterolemia effect in 40% CR and rapamycin  - Simvastatin decreased Ras protein isoprenylation-mediated reduction of growth factor receptor signaling pathways – mediated lifespan extension in Drosophila (based on Spindler et al, 2012)  - Inhibited AT1R signaling- mediated NAD(P)H oxidase inactivation and decrease reactive oxygen species level | Increased triglycerides (70.7 vs. 40.8 mg/dL) |  |  |
|  |  |  |  |  | Ramipril + simvastatin (5.0 and 20 mg/kgBW/day) |  | Exp: 36 Con: 72 | Increased by 8.6% |  | Increased glucose level (241 vs. 128 mg/dL) Increased hemorrhagic diathesis (36 vs. 15%) |  |  |
|  |  |  |  |  | Simvastatin (20 mg/kgBW/day) |  | Exp: 35 Con: 72 | NS |  |  |  | Increased benign lung tumor (17 vs. 0%) |
|  |  |  |  |  | Candesartan (1.0 mg/kgBW/day) |  | Exp: 36 Con: 72 | NS | - |  |  |  |
| 2 | (Spindler et al., 2012) | Wild-type Oregon-RC Drosophila Melanogaster | No | Male | Simvastatin (0,024 mM) | 0 days | Exp: 50 Con: 50 | Increased by 4.8% | Simvastatin decreased Ras protein isoprenylation-mediated reduction of growth factor receptor signaling pathways – mediated lifespan extension in Drosophila |  |  |  |
|  |  |  |  |  | Simvastatin (0,24 mM) |  |  | Increased by 25.0% |  | Decreased incidence of arrhythmia (arrhythmia index 0.19 vs. 0.39)  Increased in the relative time the heart spends in contraction (0.3 vs. 0.26 sec) |  |  |
|  |  |  |  |  | Simvastatin (2,4 mM) |  |  | Increased by 6.3% |  |  |  |  |
|  |  |  |  |  | Simvastatin (12 mM) |  |  | Increased by 9.1% |  |  |  |  |
| 3 | (Spindler et al., 2014) | C3B6F1 mice | No | Male | Omega-3 PUFA (467 mg/kgBW/day) | 12 months | Exp: 35 Con: 36 | NS (tend to decrease lifespan) | anticoagulant effects- mediated hemorrhagic risk and suppress CD8+ activation- mediated tumor progression | Increased hemorrhagic diathesis (42 vs 11%) |  | Increase blood urea nitrogen (19.1 vs. 14.5 mg/dL) Increased enlarged seminal vesicles (20 vs. 3%) |
| 4 | (Spindler et al., 2013) | C3B6F1 mice | No | Male | Metoprolol (1.1 g/kg diet) | 12 months | Exp: 36 Con: 36 | Increased by 9.8% | blocking beta-adrenergic receptors may decrease the G Proteins stimulation and reduce PKA activity |  |  | Increased PKA level in brain Reduced PKA level in heart Decreased mean mass of individual tumors (0.52 vs. 1.12 g) |
|  |  |  |  | Male | Nebivolol (0.27 g/kg diet) |  |  | Increased by 6.4% |  |  |  |  |
|  |  | Wild-type Oregon-RC Drosophila Melanogaster | No | Male | Metoprolol (1.1 g/kg diet) | 0 days | Exp: 200  Con: 200 | Increased by 23% |  |  |  |  |
|  |  |  |  | Male | Nebivolol (0.27 g/kg diet) |  |  | Increased by 15% |  |  |  |  |
| 5 | (Smith et al., 2019) | C3D2F1/J mice | No | Male | Acarbose (1000 ppm) | 8 months | Exp: 36 Con: 36 | Increased by 17.5% | Change of Microbiome and fecal Short-Chain Fatty Acids (SCFA) produced by gut microbial after Acarbose treatment |  |  | Increased fecal propionate level (2.7 vs. 1.4 μmols/g) Increased fecal glucose level |
|  |  | CByB6F1/J mice |  | Female |  |  |  | Increased by 4.7% |  |  |  | Increased fecal propionate level (1.9 vs. 1.0 μmols/g) Increased fecal glucose level |
| 6 | (Smith et al., 2010) | F344 rats | No | Male | Metformin (300 mg/kgBW/day) | 6 months | Exp: 45 Con: 45 | NS | - | Lowered body weight |  |  |
| 7 | (Song et al., 2019) | Wildtype silkworm strain Dazao | No | Male | Metformin (0.1 mM) | 0 days | Exp: 54 Con: 54 | Increased by 2.7% | increases antioxidant activities by regulating AMPK-P53- FoxO pathway | Decreased cocoon weight  Decreased cocoon shell ratio |  | Decreased fecundity Increased survival in starvation |
|  |  |  |  | Female | Metformin (0.1 mM) |  | Exp: 30 Con: 30 | NS | - | Decreased cocoon shell ratio |  | Decreased fecundity Increased survival in starvation |
| 8 | (Strong et al., 2016) | UM-HET3 mice | No | Male | Metformin (1000 ppm) | 9 months | Exp: 148 Con: 294 | NS | - |  |  |  |
|  |  |  |  | Female |  |  | Exp: 140 Con: 281 | NS | - |  |  |  |
|  |  |  |  | Male | Metformin (1000 ppm) + Rapamycin (14 ppm) | 9 months | Exp: 158 Con: 294 | Increased by 22.9% | metformin improves glucose homeostasis by enhancing the insulin sensitivity that perturbated by rapamycin |  |  |  |
|  |  |  |  | Female |  |  | Exp: 142 Con: 281 | NS | - |  |  |  |
|  |  |  |  | Male | Acarbose (1000 ppm) | 16 months | Exp: 147 Con: 283 | Increased by 6.3% | Not specifically discussed |  |  |  |
|  |  |  |  | Female |  |  | Exp: 135 Con: 278 | NS | - |  |  |  |
| 9 | (Harrison et al., 2014) | UM-HET3 mice | No | Male | Acarbose (1.000 ppm) | 4 months | Not available | Increased by 22% | increases FGF21 and reduces IGF-1 levels in plasma | Higher fasting blood glucose Reduced IGF1 level Reduced fasting insulin level Reduced body weight |  | - |
|  |  |  |  | Female |  |  |  | Increased by 5% |  | Higher fasting blood glucose Reduced IGF1 level Reduced body weight |  |  |
| 10 | (Harrison et al., 2019) | UM-HET3 mice | No | Male | Acarbose (400 ppm) | 8 months | Exp: 147 Con: 273 | Increased by 11% | Not specifically discussed, it sought that Acarbose increases FGF21 and reduces IGF-1 levels in plasma |  |  |  |
|  |  |  |  | Male | Acarbose (1000 ppm) |  | Exp: 161 Con: 273 | Increased by 17% |  | Reduced blood glucose after refeeding |  | Reduced adrenal medullary vasodilation Reduced liver degeneration Reduced lung tumours |
|  |  |  |  | Male | Acarbose (2500 ppm) |  | Exp: 163 Con: 273 | Increased by 16% |  |  |  |  |
|  |  |  |  | Female | Acarbose (400 ppm) |  | Exp: 139 Con: 287 | NS |  |  |  |  |
|  |  |  |  | Female | Acarbose (1000 ppm) |  | Exp: 142 Con: 287 | Increased by 5% |  | Reduced body weight  Reduced body fat |  | Reduced glomerulonecrosis Better rotarod test performance |
|  |  |  |  | Female | Acarbose (2500 ppm) |  | Exp: 152 Con: 287 | Increased by 4% |  |  |  |  |
| 11 | (Harrison et al., 2021) | UM-HET3 mice | No | Male | Candesartan (30 ppm) | 8 months | Exp: 156 Con: 303 | NS | - | - |  | - |
|  |  |  |  | Female | Candesartan (30 ppm) |  | Exp: 136 Con: 304 | NS | - |  |  |  |
| 12 | (Hasegawa et al., 2013) | Klotho−/− mice | Yes, premature aging mice | Male | Linagliptin (0.083 g/kg diet) | 5 weeks | Exp: 15 Con: 15 | Increased by 40% (p = 0.08) | Enhances phosphor-Akt, eNOS, and CREB activity in the brain | Increased body weight (11.1 ± 0.3 vs 9.9 ± 0.3 g) Increased blood glucose level (11.1 ± 0.80 vs 7.03 ± 0.46 mg/dl) Increased non-fasting blood glucose levels (183.0 ± 22.6 vs 131.3 ± 6.1 mg/dl) Lesser hypoglycemia | Increased gastrocnemius muscle mass/tibia length (3.4 ± 0.1 vs 2.9 ± 0.1 mg/mm) Increased diameter of gastrocnemius muscle (12.5 ± 0.42 vs 10.6 ± 0.63 μm) Increased kidney weight/tibia length (5.2 ± 0.2 vs 4.6 ± 0.2 mg/mm) | Increased latency in passive avoidance test Increased cerebral blood flow Increased neuronal cell number in hippocampal CA1 region Prevent alopecia |
| 13 | (Huang et al., 2017) | *C. elegans* worms*, glp-1* mutant | Yes, mutation in meiosis/mitosis decision | NA | Aspirin (100 μM) | 0 days | Exp: 585 Con: 573 | NS | - | Increased lipid hydrolysis and reduced fat storage | - | - |
| 14 | (Ito et al., 2021) | *C. elegans worms* | No | NA | Metolazone (3 µM) | 0 days | Exp: 40 Con: 46 | Increased by 20,8% | Upregulates mitochondrial chaperone-mediated mitochondrial unfolded protein response (UPRmt) activation | - | - | - |
|  |  |  |  |  | Metolazone (10 µM) |  | Exp: 66 Con: 65 | Increased by 36,4% |  |  |  |  |
|  |  |  |  |  | Metolazone (30 µM) |  | Exp: 44 Con: 46 | Increased by 29,4% |  |  |  |  |
| 15 | (Andreas et al., 2020) | *C. elegans worms* | No | NA | Lovastatin (25 µM) | 1 day | Exp: 132 Con: 128 | Increased by 12.9% | HMG-CoA reductase inhibition by lovastatin may prevent the accumulation of aging pigment and induce Jun N-terminal Kinase (JNK-1)- mediated DAF-16/Foxo longevity pathway activation | - | - | Decelerated aging pigment accumulation Increased thermal stress resistance |
|  |  |  |  |  | Lovastatin (50 µM) |  | Exp: 130 Con: 128 | Increased by 13.2% |  |  |  | Decelerated aging pigment accumulation Increased thermal stress resistance |
|  |  |  |  |  | Lovastatin (100 µM) |  | Exp: 123 Con: 128 | Increased by 24.5% |  |  |  | Decelerated aging pigment accumulation Increased thermal stress resistance |
| 16 | (Jia et al., 2022) | *C. elegans worms* | No | NA | Pioglitazone (0.1 mM) | "young adult worms" | Exp: 111 Con: 94 | Increased by 9,3% | - Activates DAF-16/FOXO- and SKN- 1/NRF2 Signaling Pathways  - Inhibits insulin/insulin-like signaling (IIS) and reproductive signaling pathways  - Activates Restriction Diet signaling pathway.  - Enhances the antioxidant activity |  |  |  |
|  |  |  |  |  | Pioglitazone (0.5 mM) |  | Exp: 138 Con: 94 | Increased by 17.8% |  | - | Extended period of fast movement | - |
|  |  |  |  |  | Pioglitazone (2 mM) |  | Exp: 74 Con: 95 | NS | - |  |  |  |
| 17 | (Santos et al., 2009) | Wistar rats | No | Male | Enalapril (10 mg/kgBW/day) | 30 days | Exp: 10 Con: 10 | Increased lifespan, percentage can not be calculated due to inadequate period of follow-up | Reduces leptin level, decrease ACE activity, and enhance Peroxisome Proliferator Activated Receptor Gamma (PPAR-γ), adiponectin, hormone-sensitive lipase, fatty acid synthase, catalase and superoxide dismutase expression levels | Decreased body weight gain Lower mean blood pressure | - | Decreased leptin levels Reduced adipose tissue ACE activity Increased the levels of PPAR-gamma, adiponectin, HSL and FAS mRNA in epididymal adipose tissue Increased gene expression of the antioxidant enzymes catalase, Cu/Zn-SOD and Mn-SOD |
| 18 | (Leibrok et al, 2015) | Klotho-hypomorphic (kl/kl) mice | Yes, premature aging mice | Male | Acetazolamide | 9 weeks | Exp: 8  Con: 9 | Increased by 201% | inhibits osteoinductive signaling, ameliorates calcification markers, and reduces aldosterone and ADH levels in Klotho-hypomorphic mice (kl/kl) mice. |  |  | Reducing blood pH, increasing blood pCO2, increasing blood Cl- |
| 19 | (Slack et al., 2012) | *Drosophila melanogaster* | No | Male | Metformin (1 mM) | 0 days | Exp: 96 Con: 96 | NS | overactivity of AMPK signaling may induce starvation-like phenotype and intestinal fluid imbalance |  |  |  |
|  |  |  |  | Male | Metformin (2.5 mM) | 0 days | Exp: 97 Con: 96 | NS |  |  |  |  |
|  |  |  |  | Male | Metformin (5 mM) | 0 days | Exp: 96 Con: 96 | NS |  |  |  |  |
|  |  |  |  | Male | Metformin (10 mM) | 0 days | Exp: 93 Con: 97 | NS |  |  |  |  |
|  |  |  |  | Male | Metformin (25 mM) | 0 days | Exp: 93 Con: 97 | NS |  |  |  |  |
|  |  |  |  | Male | Metformin (50 mM) | 0 days | Exp: 98 Con: 97 | NS |  |  |  |  |
|  |  |  |  | Male | Metformin (100 mM) | 0 days | Exp: 99 Con: 97 | Decreased by 31.5% |  |  |  |  |
|  |  |  |  | Female | Metformin (1 mM) | 0 days | Exp: 96 Con: 96 | NS |  |  |  |  |
|  |  |  |  | Female | Metformin (2.5 mM) | 0 days | Exp: 99 Con: 96 | NS |  |  |  |  |
|  |  |  |  | Female | Metformin (5 mM) | 0 days | Exp: 91 Con: 96 | NS |  |  |  |  |
|  |  |  |  | Female | Metformin (10 mM) | 0 days | Exp: 98 Con: 96 | NS |  | Decreased in triglycerides level |  |  |
|  |  |  |  | Female | Metformin (25 mM) | 0 days | Exp: 101 Con: 96 | Decreased by 10.7% |  |  |  |  |
|  |  |  |  | Female | Metformin (50 mM) | 0 days | Exp: 91 Con: 96 | Decreased by 21.5% |  |  |  | Laid fewer egg |
|  |  |  |  | Female | Metformin (100 mM) | 0 days | Exp: 99 Con: 96 | Decreased by 66.1% |  | Decreased in triglycerides level |  | Laid fewer egg |
| 20 | (Kumar et al., 2016) | *C. elegans worms* | No | NA | Captopril (1.9 mM) | 0 days | Exp: 35 Con: 48 | NS | increases stress resistance agains oxidative and heat stress and activates longevity signaling pathways |  |  |  |
|  |  |  |  |  | Captopril (2.5 mM) | 0 days | Exp: 58 Con: 48 | Increased by 34% |  |  |  |  |
|  |  |  |  |  | Captopril (3.2 mM) | 0 days | Exp: 54 Con: 48 | Increased by 18% |  |  |  |  |
| 21 |  |  |  |  | Chlorpropamide (25 µmol/L) |  | Exp: 92 Con: 105 | Extended the lifespan, percentage not specified. Chlorpropamide (400 µmol/L) had the best lifespan potential effect | Sulfonylurea (chlorpropamide) increases the mitochondrial electrical potential and SDH activity in Complex II, and mitochondrial reactive oxygen species (mtROS) |  |  |  |
|  |  |  |  |  | Chlorpropamide (100 µmol/L) |  | Exp: 88 Con: 105 |  |  |  |  |  |
|  |  |  |  |  | Chlorpropamide (400 µmol/L) |  | Exp: 111 Con: 105 |  |  |  |  |  |
|  | (Mao Z. et al., 2021) | *C. elegans worms* | No | NA | Glibenclamide (25 µmol/L) | 0 days | Exp: 58 Con: 53 | Extended the lifespan, percentage not specified. All dosage increase lifespan equally |  |  |  |  |
|  |  |  |  |  | Glibenclamide (100 µmol/L) |  | Exp: 57 Con: 53 |  |  |  |  |  |
|  |  |  |  |  | Glibenclamide (400 µmol/L) |  | Exp: 59 Con: 53 |  |  |  |  |  |
|  |  |  |  |  | Glimepiride (25 µmol/L) |  | Exp: 58 Con: 53 | Extended the lifespan, percentage not specified. Glimepiride (25 µmol/L) had the best lifespan potential effect |  |  |  |  |
|  |  |  |  |  | Glimepiride (100 µmol/L) |  | Exp: 60 Con: 53 |  |  |  |  |  |
|  |  |  |  |  | Glimepiride (400 µmol/L) |  | Exp: 59 Con: 53 |  |  |  |  |  |
| 22 | (Montalvo et al., 2013) | C57BL/6 mice | No | Male | Metformin (0.1% w/w) | 54 weeks | Exp: 64  Con: 83 | Increased by 5.8% | metformin improves antioxidant defence and increases AMP-activated protein kinase activity leading to decreasing the accumulation of oxidative stress and chronic inflammation. | Decreased body weight | Increased time to fall from an accelerating rotarod Increased treadmill performance Increased average speed | Delayed the onset of age-related cataracts |
|  |  |  |  |  | Metformin (1% w/w) |  | Exp: 90  Con: 88 | Decreased by 14.4% | Metformin at higher concentrationss induces kidney failure | Increased renal failure Increased lactic acidosis |  |  |
| 23 | (Liu W. et al., 2020) | *C. elegans* | No | NA | Verapamil (100 μM) | 0 days | Exp: 90 Con:74 | Increased by 20.6% | reduces calcineurin gene expression level and activates LGG-1/LC3 expression levels as the autophagy-associated genes | Inhibited calcineurin activity and activated autophagy mechanism, promoted autophagy processes downstream of calcineurin |  |  |
|  |  |  |  |  | Verapamil (400 μM) |  | Exp: 77 Con:74 | Increased by 19.5% |  | Inhibited calcineurin activity and activated autophagy mechanism, promoted autophagy processes downstream of calcineurin |  |  |
| 24 | (Dehghan et al., 2019) | WT C. elegans | No | NA | Hydralazine (4 days, 100 µM) | 10 days | Exp: 68 Con: 65 | Increased by 20.0% | Increases cAMP-dependent protein kinase (PKA) activity and activate SIRT1/SIR-2.1 and NRF2/SKN-1 pathway mediated mitochondrial function improvement | Improved metabolic homeostasis via the SIRT1/SIRT5 axis Improved mitochondrial function | Improved locomotor performance |  |
| 25 | (Dehghan et al., 2017) | WT C. elegans | No | NA | Hydralazine (3 days, 10 µM) | L1 larvae | Exp: 108 Con: 105 | NS | NRF2/SKN-1 signaling pathway-mediated stress resistance against oxidative stress |  |  |  |
|  |  |  |  |  | Hydralazine (3 days, 50 µM) |  | Exp: 98 Con: 86 | Increased by14.8% |  |  |  |  |
|  |  |  |  |  | Hydralazine (3 days, 100 µM) |  | Exp: 112 Con: 105 | Increased by 25% |  | Increased the localization of SKN-1 in the intestinal nuclei | Improved locomotor performance | Increased the localization of SKN-1 in the ASI neurons Complete reversal of rotenone cytotoxicity |
| 26 | (Egan et al., 2023) | WT C. elegans | No | NA | Captopril (0.3 mM) | L4 larvae (0 days) | Exp = 25 Con = 136 | NS | inhibits ACN-1 (ACE homologue for C.Elegans)- mediated dauer formation pathways activation |  |  |  |
|  |  |  |  |  | Captopril (1.6 mM) |  | Exp = 63 Con = 136 | Increased by 13% |  |  |  |  |
|  |  |  |  |  | Captopril (2.5 mM) |  | Exp = 27 Con = 136 | Increased by 33.9% |  | Reduced ACN-1 activity (worm homolog of ACE) which promoted dauer larvae formation |  | Controled aging by modulating dauer formation pathways |
|  |  |  |  |  | Captopril (3.8 mM) |  | Exp = 33 Con = 136 | Increased by 27.1% |  |  |  |  |
|  |  |  |  |  | Captopril (7.6 mM) |  | Exp = 27 Con = 136 | Decreased by 16.4% | Not specifically discussed |  |  |  |
| 27 | (Espada et al., 2020) | WT C. elegans | No | NA | Metformin (10 mM) | L4 (1 days) | Exp: 121 Con: 137 | Increased by 7.1% | Metformin activates longevity-associated pathways in young organism |  |  |  |
|  |  |  |  |  | Metformin (25 mM) |  | Exp: 129 Con: 137 | Increased by 28.5% |  |  |  |  |
|  |  |  |  |  | Metformin (50 mM) |  | Exp: 127 Con: 137 | Increased by 35.7% |  |  |  |  |
|  |  |  |  |  | Metformin (10 mM) | L4 (4 days) | Exp: 138 Con: 127 | NS |  |  |  |  |
|  |  |  |  |  | Metformin (25 mM) |  | Exp: 133 Con: 127 | NS |  |  |  |  |
|  |  |  |  |  | Metformin (50 mM) |  | Exp: 137 Con: 127 | Increased by 28.5% |  |  |  |  |
|  |  |  |  |  | Metformin (10 mM) | L4 (8 days) | Exp: 132 Con: 127 | NS |  |  |  |  |
|  |  |  |  |  | Metformin (25 mM) |  | Exp: 142 Con: 127 | NS |  |  |  |  |
|  |  |  |  |  | Metformin (50 mM) |  | Exp: 132 Con: 127 | NS |  |  |  |  |
|  |  |  |  |  | Metformin (10 mM) | L4 (10 days) | Exp: 125 Con: 137 | Decreased by 21.4% | Metformin induces ATP exhaustion- mediated mitochondrial dysfunction and respiratory failures in late life organism | Metformin toxicity is associated with mitochondrial dysfunction and reduction of mitochondrial mass in ageing |  |  |
|  |  |  |  |  | Metformin (25 mM) |  | Exp: 130 Con: 137 | Decreased by 21.4% |  |  |  |  |
|  |  |  |  |  | Metformin (50 mM) |  | Exp: 134 Con: 137 | Decreased by 21.4% |  |  |  |  |
| 28 | (Preuss et al., 2011) | Zucker Fatty Rats | No | Male | Niacin-bound chromium (NBC) | 8 weeks | Exp: 12 Con: 10 | Increased by 14.1% | - | Lower systemic blood pressure on treatment groups vs. controls Lower blood glucose on treatment groups vs. control |  |  |
| 29 | (Strong et al.,2008) | UM-HET3 mice | No | Male | Aspirin (3.3 mg/kgBW/day) | 4 months | Exp: 132 Con: 264 | Increased by 8.0% | Not specifically analyzed, it sought that combination Nordihydroguaiaretic acid (NDGA) and aspirin have antioxidant and anti-inflammation activities |  |  |  |
|  |  |  |  | Female |  |  | Exp: 108 Con: 216 | NS |  |  |  |  |
| 30 | (Strong et al., 2022) | UM-HET3 mice | No | Male | Captopril (180 ppm) | 5 months | Exp: 150 Con: 285 | Increased by 14.3% | - | Lower body weight at 12 and 18 months |  |  |
|  |  |  |  | Female |  |  | Exp: 132 Con: 276 | Increased by 5.4 % |  | Lower body weight at 12, 18 and 24 months |  |  |
|  |  |  |  | Male | Acarbose (1000 ppm) + rapamycin (14.7 ppm) | 9 months | Exp: 153 Con: 285 | Increased by 36.6% | it sought that acarbose induces insulin sensitivity after rapamycin treatment. Additionally, acarbose alters changes in the gut microbiome and increases short-chain fatty acids (SCFA) levels | Lower body weight at 12, 18 and 24 months |  |  |
|  |  |  |  | Female |  |  | Exp:134 Con: 276 | Increased by 27.5% |  | Lower body weight at 12, 18 and 24 months |  |  |
|  |  |  |  | Male |  | 16 months | Exp: 138 Con: 285 | Increased by 13.5% |  | Lower body weight at 18 and 24 months |  |  |
|  |  |  |  | Female |  |  | Exp: 134 Con: 276 | Increased by 12.4% |  | Lower body weight at 24 months |  |  |
| 31 | (Xiao et al., 2022) | C.elegans wild type N2 | No | NA | Metformin (50mM) | L4 larvae (0 days) | Exp: 100 Con: 100 | Increased by 26.3% | decrease in S-Adenosylmethionine (SAM) levels-mediated H3K4me3 modifiers inhibition and alters mTOR/RSKS-1 signaling in adult worms |  |  | Reduced S-adenosylmethionine (SAM) level Downregulating mTOR and S6 kinase |
| 32 | (Xu et al., 2020) | C57BL/6J mice | No | Male | Rosiglitazone (1mg/kgBW/day) | 14 months | Exp: 34 Con: 31 | Increased by 11.2% | reduces inflammation and preserves mitochondrial function in adipose, liver, muscle, and brain tissues | Improved insulin sensitivity and prevent adipose tissue loss in aging mice |  | reduces inflammation, fibrosis, and atrophy in aging tissues Relieves anxiety- and depression-like symptoms and improves cognitive functionality |
| 33 | (Zhu et al., 2021) | c57BL/6 mice | No | Female | Metformin (100mg/kgBW/day) | 20 months | Exp: 21 Con: 31 | Decreased by 1.5% | Supressing mitochondrial respiration, lowering ATP production, and induce metabolic reprogramming by activating glycolysis, but enhances the extracellular matrix-related gene in the heart |  |  | increase cardiac stress markers in metformin group (Myh7/Myh6, Nppa, Nppb) |
| 34 | (Wan et al., 2013) | C.elegans wild type N2 | No | NA | Aspirin (50 µM) | L4 larvae (0 days) | Exp: 160 Con: 123 | Increased by 8.2% | activates ampk and DAF-16/FOXO signaling pathway |  | Increased fast body movement |  |
|  |  |  |  |  | Aspirin (100 µM) |  | Exp: 543 Con: 341 | Increased by 15.5% |  |  | Increased fast body movement |  |
|  |  |  |  |  | Aspirin (200 µM) |  | Exp: 185 Con: 123 | Increased by 12.5% |  |  | Increased fast body movement |  |
| 35 | (Yang et al., 2019) | C.elegans wild type N2 | No | NA | Nicotinic Acid (100 nmol) | 0 days | Exp: 90 Con 90 | NS | - |  |  |  |
|  |  |  |  |  | Nicotinic Acid (200 nmol) |  |  | NS | - |  |  |  |
|  |  |  |  |  | Nicotinic Acid (600 nmol) |  |  | Increased by 17.5% | Increase the intracellular nicotinamide adenine dinucleotide (NAD+) levels but sould be lower than the Sirtuin- NAD+ saturated concentration |  |  |  |
| 36 | (Miller et al. 2020) | UM-HET3 mice | No | Male | Canaglifozin (180 ppm) | 7 months | Exp: 156 Con: 303 | Increased by 13.9% | Not specifically discussed, it sought that canaglifozin enhances fatty acids and ketones metabolism, suppress TORC1 signaling pathway and increases AMPK activity in liver tissue. Canaglifozin may also increase FGF21 levels in the serum | Lower blood glucose |  |  |
|  |  |  |  | Female |  |  | Exp: 136 Con: 304 | NS | - | Lower body weight Lower fat mass Lower blood glucose |  |  |
| 37 | (Abrat et al., 2018) | Drosophila melanogaster | No | Male | Metformin (10 mM) | 5 days | Exp: 300 Con: 294 | Decreased by 25.0% | It was not directly related to metformin. Probably the starch diet that was used in this study disrupted metabolic homeostasis and induced a shortened lifespan in the flies. |  |  |  |
|  |  |  |  | Female |  |  | Exp: 296 Con: 282 | Decreased by 39.3% |  |  |  |  |
| 38 | (Ayyadevara et al., 2013) | C. elegans | No | NA | Aspirin (0.5 mM) | L4 | Exp: 166 Con: 162 | Increased by 23.0% | Inhibits oxidant stress and activates antioxidant defense mechanism |  |  | Decreased aging-associated aggregation Reduced reactive oxygen species |
|  |  |  |  |  | Aspirin (1 mM) |  | Exp: 158 Con: 162 | Increased by 20.9% |  |  |  | Decreased pharyngeal pumping Decreased aging-associated aggregation Reduced reactive oxygen species |
| 39 | (Banse et al., 2023) | C. elegans | No | NA | Acarbose (500 µM) | L4 (1 days) | Exp: 300 Con: 300 | NS | - |  |  |  |
|  |  | C. briggsae |  |  |  |  | Exp: 300 Con: 300 |  |  |  |  |  |
|  |  | C. tropicalis |  |  |  |  | Exp: 300 Con: 300 |  |  |  |  |  |
| 40 | (Anisimov et al., 2015) | 129/Sv mice | No | Male | Metformin (100 mg/kgBW/day) | First 7 days of life | Exp: 39 Con: 50 | Increased by 20.0% | Not specifically discussed, it sought that metformin increased AMPK and antioxidant activities | Decrease in bodyweight |  |  |
|  |  |  |  | Female |  |  | Exp: 30 Con: 35 | Decreased by 9.1% | - | Decrease total cholesterol level |  | Reduced fraction of mice with regular estrous cycles Increased MDA level |
| 41 | (Brandstädt et al., 2013) | C. elegans N2 (wild type) | No | NA | Clofibrate (0.1 µM) | L4 | Exp: 150 Con: 1650 | NS |  |  |  |  |
|  |  |  |  |  | Clofibrate (1 µM) |  | Exp: 150 Con: 1650 | NS |  |  |  |  |
|  |  |  |  |  | Clofibrate (10 µM) |  | Exp: 450 Con: 1650 | Increased by 16.2% | activates NHR-49 (homologue for Peroxisome Proliferator‐Activated Receptor Alpha (PPAR-α) in mammal)-mediating mitohormesis promotion |  |  |  |
|  |  |  |  |  | Fenofibrate (0.1 µM) |  | Exp: 150 Con: 1650 | Increased by 7.9% |  |  |  |  |
|  |  |  |  |  | Fenofibrate (1 µM) |  | Exp: 150 Con: 1650 | Increased by 5.5% |  |  |  |  |
|  |  |  |  |  | Fenofibrate (10 µM) |  | Exp: 450 Con: 1650 | Increased by 18.5% |  |  |  |  |
| 42 | (Cabreiro et al., 2013) | C. elegans N2 (wild type) | No | NA | Metformin (25 mM) | L4 (0 days) | Not available | Increased by 18.3% | Metformin inhibits the metabolism of folate and methionine in E.Coli, which serves as a food supply for C.elegans. |  |  |  |
|  |  |  |  |  | Metformin (50 mM) |  |  | Increased by 36.3% |  |  |  |  |
|  |  |  |  |  | Metformin (100 mM) |  |  | NS |  |  |  |  |
|  |  |  |  |  | Metformin (25 mM) | L4 (8 days) |  | Increased by 8% |  |  |  |  |
|  |  |  |  |  | Metformin (50 mM) |  |  | NS |  |  |  |  |
|  |  |  |  |  | Metformin (100 mM) |  |  | NS |  |  |  |  |
| 43 | (Onken et al., 2010) | C. elegans N2 (wild type) | No | NA | Metformin (1 mM) | L4 (0 days) | Exp: 290 Con: 554 | Increased by 13,3% | Activates AMPK, LKB1, and SKN-1 signaling pathways |  |  |  |
|  |  |  |  |  | Metformin (10 mM) |  | Exp: 324 Con: 554 | NS |  |  |  | Decreased nile red fluorescence |
|  |  |  |  |  | Metformin (50 mM) |  | Exp: 521 Con: 554 | Increased by 26,6% |  |  |  | Decreased age pigment fluorescence Decreased nile red fluorescence Decreased average body bend / 30 second in 10 and 15 days |
| 44 | (Champigny et al., 2018) | Drosophila melanogaster | No | Male | Omega 3 (40.08% EPA; 0.3 mg/mL) | 0 days | Exp: >145 Con: >145 | Increased by 14.6% | Increases the antioxidant enzyme (superoxide dismutase) activity, delays lipid peroxidation process, and preserves mitochondrial metabolism |  |  | Decreased mitochondrial proton leak Higher electron transport system capacity Higher mitochondrial oxidative capacity Maintenance of superoxide dismutase activity Delayed the occurrence of lipid peroxidation |
|  |  |  |  | Male | Omega 3 (40,72% DHA; 0.3 mg/mL) |  | Exp: >145 Con: >145 | Increased by 14.6% |  |  |  | Decreased mitochondrial proton leak Delayed the occurrence of lipid peroxidation |
| 45 | (Cedillo et al., 2023) | C. elegans N2 (wild type) | No | NA | Metformin (50 mM) | L4 (0 days) | Exp: 140 Con: 150 | Increased by 38.5% | Regulates ether lipid biogenesis |  |  |  |
| 46 | (Chen et al., 2017) | C. elegans N2 (wild type) | No | NA | Metformin (50 mM) | L4 (0 days) | Exp: 300 Con: 300 | Extended the lifespan, percentage not specified. | Regulates v-ATPase-Ragulator-AXIN/LKB1 of the lysosomal pathway- mediated mTORC1 inhibition and AMPK activation | Decreased neutral fat level | Increased the locomotory ability (average bends of worm body per 60 s) | Reduced age pigments |
| 47 | (Danilov et al., 2015) | wild-type strain Canton-S Drosophila Melanogaster | No | Male | Aspirin (0.05 µM) | 0 days | Exp: 300 Con: 300 | Increased by 13% | downregulates Pkh2-ypk1-lem3-tat2 pathway |  |  |  |
|  |  |  |  |  | Aspirin (0.5 µM) |  | Exp: 300 Con: 300 | Increased by 6% |  |  | Decreased spontaneous activity Increased the activity of negative geotaxis test |  |
|  |  |  |  |  | Aspirin (1 µM) |  | Exp: 300 Con: 300 | Increased by 7% |  |  | Decreased spontaneous activity Increased the activity of negative geotaxis test |  |
|  |  |  |  | Female | Aspirin (0.05 µM) | 0 days | Exp: 300 Con: 300 | Increased by 12% |  |  |  | Increased number of egg Decreased number of pupae |
|  |  |  |  |  | Aspirin (0.5 µM) |  | Exp: 300 Con: 300 | Increased by 8% |  |  | Increased the activity of negative geotaxis test | Decreased number of eggs |
|  |  |  |  |  | Aspirin (1 µM) |  | Exp: 300 Con: 300 | Increased by 33% |  |  |  | Decreased number of eggs |
| 48 | (De Haes et al., 2014) | C. elegans N2 (wild type) | No | NA | Metformin (50 mM) | L1 | Exp: 92 Con: 97 | Increased by 47% | regulates mitohormesis by increasing mitochondrial respiration-mediated oxidative stress production and activates antioxidant peroxiredoxin PRDX-2 | Maintain worm volume | Decreased BCAA contraction Decreased olil Red O intensity | Increased metabolic heat production Increased respiration Increased H2O2 release Increased PRDX-2 disulfides |
|  |  |  |  |  |  | L1-L4 only | Exp: 99 Con: 97 | NS |  |  |  |  |
|  |  |  |  |  |  | Adult phase only | Exp: 102 Con: 97 | Increased by 57% |  |  |  |  |
|  |  |  |  |  |  | 5 days of adult phase only | Exp: 98 Con: 97 | Increased by 43% |  |  |  |  |
| 49 | (Onken et al., 2021) | C. elegans (N2, JU775, and MY16) | No | NA | Metformin (0-100 µM) | Adult phase (day 1) | Exp: 480 Con: 480 | Increased by 35% (50 µM); 41% (70 µM) | Metformin does not cross the cuticle due to its high polarity |  | Increased mean composite swimming score |  |
|  |  | C. briggsae (AF16, ED3092, and HK104) |  |  |  |  |  | NS | - |  | Increased mean composite swimming score |  |
|  |  | C. tropicalis (JU1373, JU1630, and QG834) |  |  |  |  |  | Decreased by 12% (10 µM); 16% (50 µM) | AMPK/SKN-1 protective mechanism is less responsive to metformin administration in C. tropicalis compared with C. elegans |  | Decreased mean composite swimming score |  |
|  |  | C. elegans (N2, JU775, and MY16) |  |  | Dapaglifozin (0-100 µM) |  |  | Decreased lifespan (100 and 300 µM) | - |  |  |  |
|  |  | C. briggsae (AF16, ED3092, and HK104) |  |  |  |  |  | Decreased lifespan (100 and 300 µM) | - |  |  |  |
|  |  | C. tropicalis (JU1373, JU1630, and QG834) |  |  |  |  |  | Decreased lifespan (300 µM) | - |  |  |  |
|  |  | C. elegans (N2, JU775, and MY16) |  |  | Sitagliptin (0-100 µM) |  |  | Increased lifepan (1000 µM) | Not specifically discussed |  |  |  |
|  |  | C. briggsae (AF16, ED3092, and HK104) |  |  |  |  |  | NS | - |  |  |  |
|  |  | C. tropicalis (JU1373, JU1630, and QG834) |  |  |  |  |  | Increased lifepan (10 µM) | Not specifically discussed |  |  |  |
|  |  | C. elegans (N2, JU775, and MY16) |  |  | Nateglinide (0-100 µM) |  |  | Decreased lifespan (1 and 5 µM) | - |  |  |  |
|  |  | C. briggsae (AF16, ED3092, and HK104) |  |  |  |  |  | NS | - |  |  |  |
|  |  | C. tropicalis (JU1373, JU1630, and QG834) |  |  |  |  |  | Decreased lifespan (100 µM) | - |  |  |  |
|  |  | C. elegans (N2, JU775, and MY16) |  |  | Pioglitazone (0-100 µM) |  |  | NS | - |  |  |  |
|  |  | C. briggsae (AF16, ED3092, and HK104) |  |  |  |  |  | NS | - |  |  |  |
|  |  | C. tropicalis (JU1373, JU1630, and QG834) |  |  |  |  |  | NS | - |  |  |  |
|  |  | C. elegans (N2, JU775, and MY16) |  |  | Glipizide (0-100 µM) |  |  | NS | - |  |  |  |
|  |  | C. briggsae (AF16, ED3092, and HK104) |  |  |  |  |  | NS | - |  |  |  |
|  |  | C. tropicalis (JU1373, JU1630, and QG834) |  |  |  |  |  | NS | - |  |  |  |

Note: blue indicates a positive effect on lifespan extension, whereas red indicates a negative effect. Exp: experimental total subject. Con: control total subject. NS: not significant

Supplementary Table 5: Search for cardiometabolic drugs in the International Clinical Trials Registry Platform (ICTRP) with potential to extend lifespan

| Drug search at ITCRP | Hits |
| --- | --- |
| Acarbose | 2 |
| Acetazolamide | 0 |
| Aspirin | 2 |
| Canaglifozin | 0 |
| Candesartan | 1 |
| Captopril | 0 |
| Chlorpropamide | 0 |
| Clofibrate | 0 |
| Dapaglifozin | 0 |
| Enalapril | 0 |
| Fenofibrate | 1 |
| Glibenclamide | 0 |
| Glimepiride | 1 |
| Glipizide | 0 |
| Hydralazine | 0 |
| Linagliptin | 0 |
| Lovastatin | 0 |
| Metformin | 17 |
| Metolazone | 0 |
| Metoprolol | 3 |
| Nateglinid | 0 |
| Nevibolol | 0 |
| Nicotinic acid | 0 |
| Omega-3 PUFA | 11 |
| Pioglitazone | 2 |
| Ramipril | 0 |
| Rosiglitazone | 0 |
| Simvastatin | 2 |
| Sitagliptin | 2 |
| Tolbutamide | 0 |
| Verapamil | 0 |

Note: the hits were counted before exclusion parameters being applied.

Supplementary table 6. Unknown, terminated, and withdrawn clinical trial registry in repurposing cardiometabolic drug for aging

| Clinical trial identifier | Year of registration | Study name | Drug name | Status |
| --- | --- | --- | --- | --- |
| NCT00627653 | 2008 | Effect of a PPAR-Alpha Agonist on the Age Related Changes in Myocardial Metabolism and Mechanical Function | Fenofibrate | Unknown |
| NCT02745886 | 2016 | Metformin Induces a Dietary Restriction-like State in Human | Metformin | Unknown |
| NCT03451006 | 2018 | Effect of Metformin on Frailty in 12 Subjects | Metformin | Terminated^*^ |
| NCT04994561 | 2022 | VIAging Deceleration Trial Using Metformin, Dasatinib, Rapamycin and Nutritional Supplements | Metformin | Withdrawn |

^*^Terminated due to recruitment being difficult and not achieved

Supplementary Table 7. Risk of bias in clinical trial studies

| **Risk of bias parameter** | **Kuzlow 2016** | **Swanson 2018** | **Kulkarni 2018** | **Fairbairn 2020** |
| --- | --- | --- | --- | --- |
| Randomisation process | 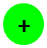 | 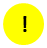 | 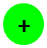 | 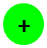 |
| Deviations from the intended interventions | 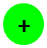 | 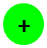 | 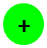 | 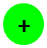 |
| Missing outcome data | 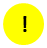 | 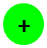 | 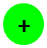 | 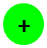 |
| Measurement of the outcome | 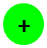 | 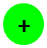 | 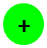 | 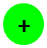 |
| Selection of the reported result | 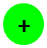 | 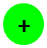 | 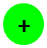 | 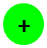 |
| **Overall** | 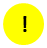 | 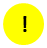 | 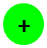 | 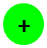 |

Note:
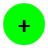
 indicating low risk of bias,
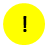
 indicating some concern of bias,
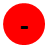
 indicating high risk of bias
